# Glyconanofluorides as Immunotracers with a Tunable Core Composition for Sensitive Hotspot Magnetic Resonance Imaging of Inflammatory Activity

**DOI:** 10.1021/acsnano.1c01040

**Published:** 2021-04-19

**Authors:** Dana Cohen, Reut Mashiach, Lothar Houben, Andrea Galisova, Yoseph Addadi, David Kain, Alisa Lubart, Pablo Blinder, Hyla Allouche-Arnon, Amnon Bar-Shir

**Affiliations:** † Department of Organic Chemistry, Weizmann Institute of Science, Rehovot 7610001, Israel; ‡ Department of Chemical Research Support, Weizmann Institute of Science, Rehovot 7610001, Israel; § Life Sciences Core Facilities, Weizmann Institute of Science, Rehovot 7610001, Israel; ∥ Neurobiology, Biochemistry and Biophysics School, George S. Wise Faculty of Life Sciences, Tel Aviv University, Tel Aviv 69978, Israel

**Keywords:** biomimetic, glyconanoparticles, nanocrystals, inflammation, ^19^F MRI, multicolor MRI

## Abstract

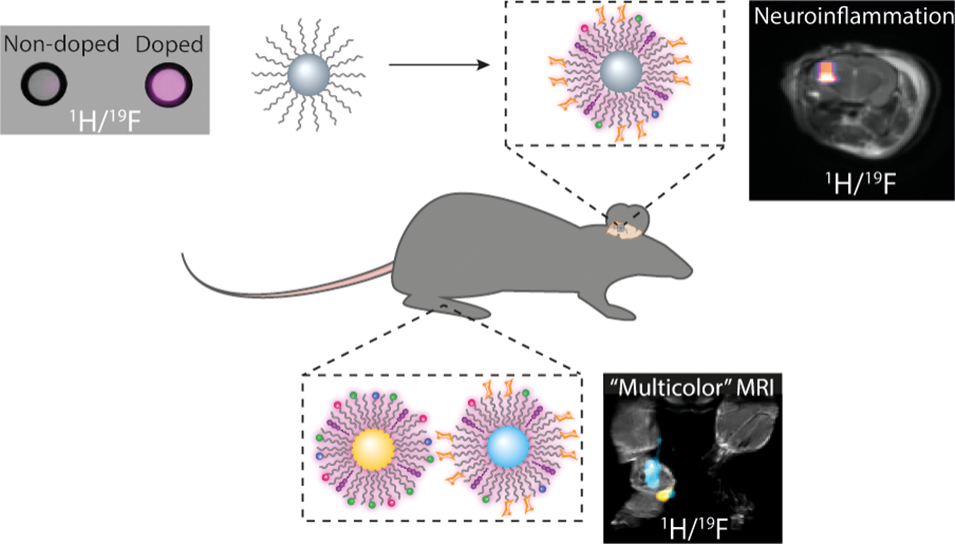

Nature-inspired nanosized formulations based on an imageable, small-sized inorganic core scaffold, on which biomolecules are assembled to form nanobiomimetics, hold great promise for both early diagnostics and developed therapeutics. Nevertheless, the fabrication of nanobiomimetics that allow noninvasive background-free mapping of pathological events with improved sensitivity, enhanced specificity, and multiplexed capabilities remains a major challenge. Here, we introduce paramagnetic glyconanofluorides as small-sized (<10 nm) glycomimetics for immunotargeting and sensitive noninvasive *in vivo*^19^F magnetic resonance imaging (MRI) mapping of inflammation. A very short *T*_1_ relaxation time (70 ms) of the fluorides was achieved by doping the nanofluorides’ solid crystal core with paramagnetic Sm^3+^, resulting in a significant 8-fold enhancement in their ^19^F MRI sensitivity, allowing faster acquisition and improved detectability levels. The fabricated nanosized glycomimetics exhibit significantly enhanced uptake within activated immune cells, providing background-free *in vivo* mapping of inflammatory activity, demonstrated in both locally induced inflammation and clinically related neuropathology animal models. Fabricating two types of nanofluorides, each with a distinct chemical shift, allowed us to exploit the color-like features of ^19^F MRI to map, in real time, immune specificity and preferred targetability of the paramagnetic glyconanofluorides, demonstrating the approach’s potential extension to noninvasive multitarget imaging scenarios that are not yet applicable for nanobiomimetics based on other nanocrystal cores.

Small (<10 nm) inorganic nanocrystals (NCs) offer well-defined solid scaffolds for the “bottom-up” engineering of nanostructured biomimetic formulations.^1^ Decorating their solid core with natural moieties, such as protein nanocages,^2^ viral capsids,^3^ spherical nucleic acids,^4^ multivalent glycans,^5^ or lipoproteins,^6^ endows these fabrications with both the structure and function of a natural nanosized formulation. Their further engineering with imageable solid cores^6−13^ adds the option of noninvasive imaging of biological processes in deep tissues of live subjects, with improved biodistribution profiles and enhanced tissue accessibility and targetability.^14^ Among the biomedical imaging modalities for which these nanostructured biomimetics have been designed, magnetic resonance imaging (MRI) stands out due to its ability to provide target-specific information from the nanostructure agent that can be accurately localized on high-resolution anatomical images of soft tissues.^15^ Moreover, the ability to obtain complementary physiological^16^ and functional^17^ information from biodegradable MRI agents makes this modality preferable for molecular imaging applications. Yet, although highly sensitive,^18^ the currently used NCs for MRI are based on metal-oxide cores that generate nonspecific, strong background signals, which are not quantifiable and cannot be mapped in a “hotspot” background-free display fashion, as demonstrated for nanoformulation designs proposed for CEST-MRI^19−21^ or heteronuclear MRI^22,23^ applications.

As an alternative to magnetic NCs used for ^1^H MRI, it was recently demonstrated that fabricated small (<10 nm) inorganic nanofluorides can serve as nanotracers for *in vivo*^19^F MRI.^24^ Combining the advantages of NCs^25^ with the benefits of ^19^F MRI,^26^ these nanofluorides offer MRI-detectable inorganic NCs that can be used as a small-sized solid scaffolds for the fabrication of nature-inspired nanomaterials. Contrary to ^1^H MRI, ^19^F MRI agents provide a platform for background-free MR signals that have the potential to be quantified as well as displayed as hotspot maps.^23,27−32^ Moreover, the ^19^F MR signal can be perfectly co-registered on high-resolution ^1^H MRI data, with the potential to be further designed and presented in a multicolor fashion,^33−36^ retarding the need for hybrid multimodal imaging technologies. Nevertheless, although nanofluorides offer the small-sized solid core needed for engineering nanobiomimetics, a feat not possible with large-sized (100–200 nm) fluorine-based emulsions, their *T*_1_ relaxation times are relatively long.^24^ This limits signal averaging and, thus, the signal-to-noise ratio (SNR) in ^19^F MR images, restricting nanofluorides’ applicability to dynamic longitudinal studies and their ability to detect low-concentration targets at a given imaging time.

Here, we demonstrate the fabrication of paramagnetic nanofluorides that possess extremely short *T*_1_ values for enhanced ^19^F MRI sensitivity, much shorter than that achieved by fabricating polycrystalline nanofluorides.^37^ Inspired by glyconanoparticles^5,38^ that display carbohydrate-based structures for improved recognition and enhanced affinity to inflammatory cells,^39−41^ we designed paramagnetic glyconanofluorides as a tunable platform for noninvasive hotspot MRI mapping of inflammation. Specifically, we demonstrate that nanofluorides (CaF_2_ or SrF_2_) doped with paramagnetic elements for enhanced ^19^F MRI sensitivity and further coated with multivalent lactose moieties can function as imageable tracers with enhanced immunotargetability. Contrary to magnetic glyconanoparticles,^42,43^ which rely on the identification and interpretation of MRI signal voids,^15,44^ glyconanofluorides offer background-free MRI signals from targeted immune cells. The fabricated paramagnetic glyconanofluorides enable specific, *in vivo*^19^F MRI mapping of inflammatory activity, both in a locally induced inflammation model and in a model of neuroinflammation. Our demonstration of enhanced immune targetability in a multicolor fashion when using two types of nanofluorides (CaF_2_ and SrF_2_) emphasizes another feature of these nanotracers, representing the potential for their further development with other targets and applications where small-sized inorganic NCs are advantageous.

## Results and Discussion

### Oleate-Coated Ln:CaF_2_ Fabrications

**Figure 1:**
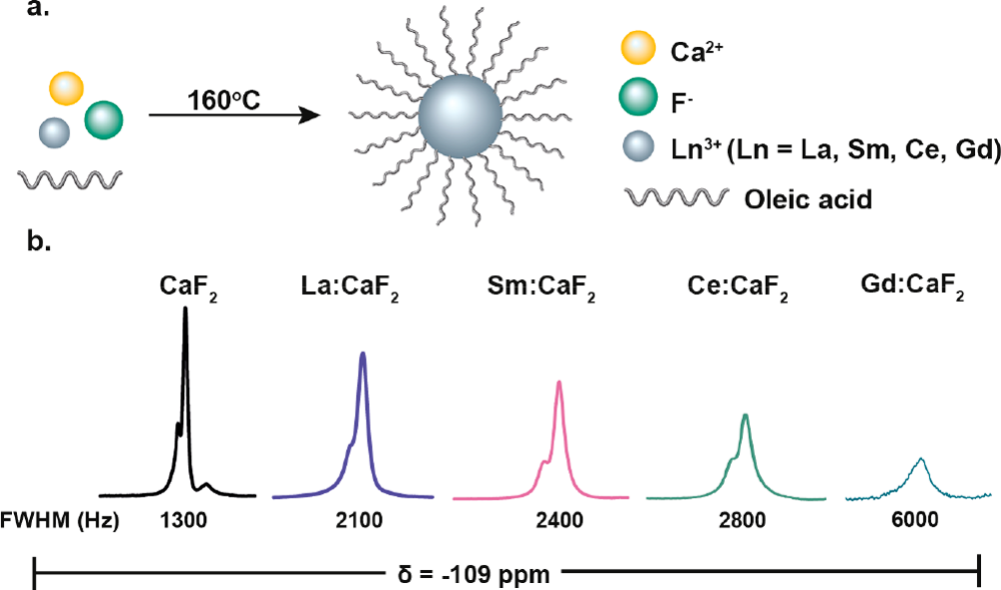
Paramagnetic OA-CaF_2_ NCs. (a) Schematic representation of the synthetic route used for the fabrication of paramagnetic OA-CaF_2_ and OA-Ln:CaF_2_ NCs. (b) ^19^F NMR spectra of OA-Ln:CaF_2_ NCs with different Ln^3+^ dopants (5 mol % in the synthesis).

We created modifiable NCs that allow both paramagnetic doping (for enhanced ^19^F MRI sensitivity) and surface functionalization (for multivalent glycan presentation) by first synthesizing small-sized CaF_2_ NCs using the phase transfer and separation synthetic approach, with oleic acid (OA) serving as the NCs’ capping ligand (Figure 1a).^45^ The obtained OA-CaF_2_ NCs were small enough (<10 nm, by dynamic light scattering (DLS), Figure S1) to average out homonuclear dipolar interactions,^24^ providing a typical high-resolution ^19^F NMR signal at −109 ppm (Figure 1b). As the very long *T*_1_ (16 s, Figure S2) of the fluoride content within OA-CaF_2_ NCs restricts their applicability for *in vivo*^19^F MRI studies, as it imposes a long data acquisition time frame, we next doped them with lanthanide cations (Ln^3+^), which have an ionic radius relatively similar to that of Ca^2+^. This allowed us to introduce paramagnetic elements that induce a paramagnetic relaxation enhancement (PRE) effect on the neighboring fluorides (F^–^) in the crystal. The Ln^3+^-doped CaF_2_ (OA-Ln:CaF_2_) fabrications were synthesized by adding calculated amounts of Ln(NO_3_)_3_ to the reaction mixture (for further details, see the Methods section). To that end, 5 mol % of Ln^3+^-doped oleate-coated CaF_2_ NCs were synthesized, purified, and characterized, resulting in a series of OA-Ln:CaF_2_ NCs (Figure 1b). In order to examine the PRE induction capabilities of the dopants, four representative Ln^3+^ cations were used: La^3+^ as a diamagnetic dopant, Sm^3+^ and Ce^3+^ as Ln^3+^ cations with the mildest PRE induction capabilities, and Gd^3+^ with the largest PRE induction capabilities. Of note, we obtained for all OA-Ln:CaF_2_ fabrications small-sized (<10 nm) monodispersed nanofluorides (DLS, Figure S1) that yielded a clearly resolved high-resolution ^19^F NMR signal while in solution (Figure 1b).

### Ln^3+^ Dopants Induce a PRE Effect in CaF_2_ NCs

The ability of a PRE induction to improve the performances of ^19^F MRI was previously demonstrated in large-sized (150–200 nm) perfluorocarbon (PFC) emulsions in which the chelated paramagnetic elements were embedded in the fluorous phase of the formulation.^46−48^ As expected, we observed a mild effect on ^19^F NMR line-broadening with the diamagnetic lanthanide, La^3+^ (calculated from the full width at half-maximum, fwhm), and no effect on the *T*_1_ relaxation properties (La:CaF_2_ compared CaF_2_; Figure 1b and Figure S2). A more pronounced line-broadening effect was, however, obtained when the paramagnetic lanthanide cations were examined (*i.e.*, Sm^3+^ Ce^3+^ and Gd^3^) as dopants. Note that the “shoulder” peak, which resonates at −105 ppm, is assigned to fluorides at the surface of the CaF_2_ NCs, while the main peak, which resonates at −109 ppm, represents the fluorides at their core.^49^ Upon the addition of dopants, these peaks tend to coalesce, probably due to NMR line-broadening but also due to a smaller deviation in the chemical environment of the fluorides in the core and the shell of the NC. This observation is in agreement with phenomenon detected when highly crystalline CaF_2_ NCs were compared to ones with a defected crystal core.^37^

**Figure 2:**
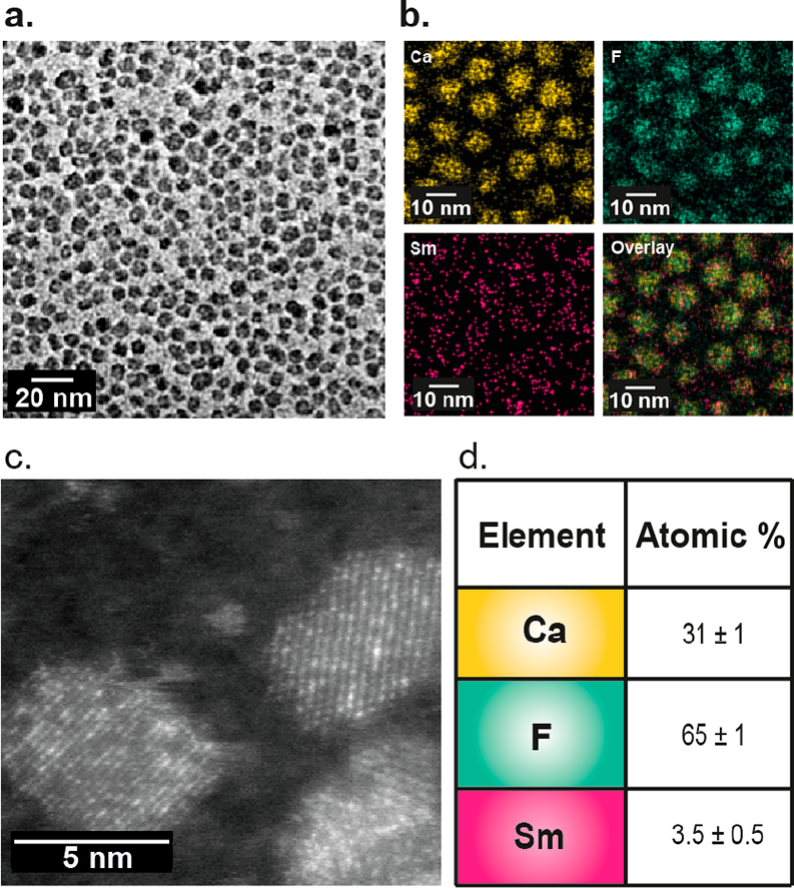
EM images and analysis of Sm-doped CaF_2_NCs. (a) TEM image of Sm:CaF_2_ NCs. (b) EDS elemental maps showing the elements’ distribution in the NCs. (c) High-resolution STEM/high-angle annular dark-field showing the Sm^3+^ ions as bright spots, depicting their incorporation into the NC lattice. (d) Quantitative analysis of the atomic ratio in Sm:CaF_2_ NCs, calculated from the EDS data.

In contrast to the pronounced ^19^F NMR line-broadening obtained upon CaF_2_ doping with Ce^3+^ and Gd^3+^, which reduces the SNR of the resultant ^19^F MRI,^46^ doping the nanofluorides with Sm^3+^ dramatically shortens the *T*_1_ relaxation time of the ^19^F content compared to that with OA-CaF_2_ or OA-La:CaF_2_ (compare inversion recovery plots, Figure S2) while having only a mild effect on ^19^F NMR line-broadening and *T*_2_ characteristics (Figure 1b and Figure S2). The very broad line and the relatively poor SNR obtained for OA-Gd:CaF_2_ did not allow us to determine its *T*_1_ and *T*_2_ values using the experiments used to evaluate these relaxation times for the other nanofluorides. These observations are in good agreement with previous reports that “NMR visibility” and, thus, the quantification of adjacent atoms in Ln^3+^-doped crystals is not significantly affected by Sm^3+^ as compared to crystals doped with lanthanides with more pronounced PRE capabilities.^50^ It should be noted here that paramagnetic dopants may reduce the NMR signal of neighboring nuclear spins due to relaxation, paramagnetic broadening, and/or shielding. When such an effect by the dopant is strong, a “blind sphere” radius at which the NMR signal of neighboring nuclei is nullified can be defined.^51^ Sm^3+^ induces a negligible blind sphere radius compared to other lanthanides;^50^ thus, although it induces a significant PRE effect in nanofluorides (Figure S2), it should allow the detection of NMR signals even from atoms that are very close to the paramagnetic center of the NC lattice. Nevertheless, increasing the amount of Sm^3+^, which does not affect the size or dispersity of the obtained Sm:CaF_2_ NCs (Figures S3–S5), does, indeed, lead to an even shorter *T*_1_ of the fluoride within the NCs but also leads to ^19^F NMR line-broadening, resulting in a poor SNR, which may cancel-out the *T*_1_-shortening gain in ^19^F MRI studies (Figure S6). The transmission electron microscopy (TEM) images of OA-Sm:CaF_2_ (Figure 2a) clearly indicate that the addition of 5% of Sm^3+^ dopant through the fabrication of OA-Sm:CaF_2_ does not affect the NC’s small size (6 ± 0.8 nm *vs* the 5.5 ± 1.3 nm obtained for OA-CaF_2_), with homogeneous distribution of the doping element deduced from energy-dispersive X-ray spectroscopy (EDS) elemental maps (Figures 2b). Notably, atomic-resolution scanning transmission electron microscope (STEM) images of a single Sm:CaF_2_ NC revealed bright spots, which are associated with the strong high-angle scattering of the heavy Sm atoms, demonstrating their incorporation into the lattice sites of the CaF_2_ crystal (Figure 2c and Figure S7). The quantification of the EDS maps allowed us to determine the atomic ratio in the Sm:CaF_2_ NCs, which was found to be (31 ± 1):(65 ± 1):(3.5 ± 0.5) for Ca:F:Sm (Figure 2d).

### Phospholipid-Coated Ln:CaF_2_ NCs

**Figure 3:**
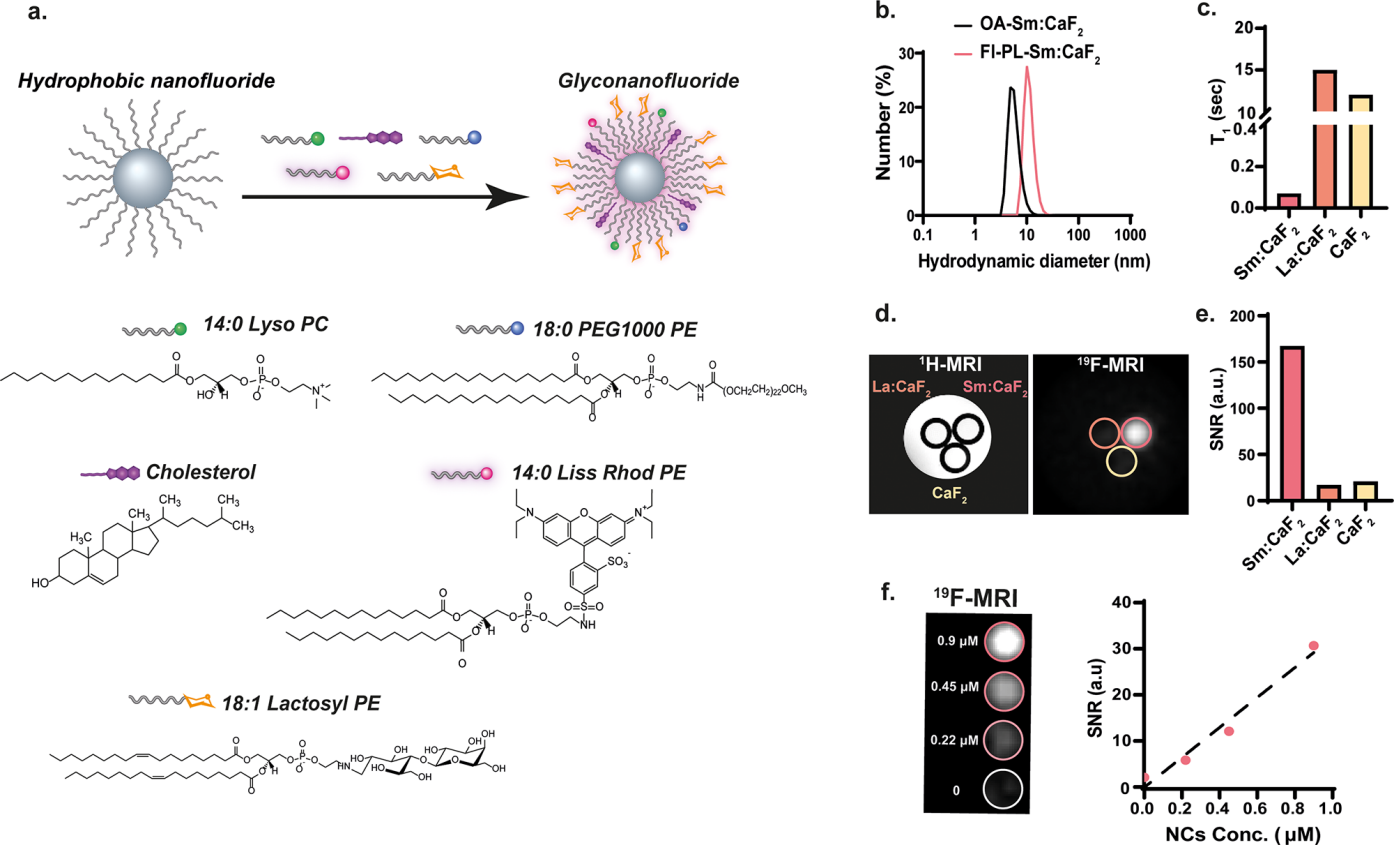
Water-soluble phospholipid-coated Sm:CaF_2_NCs (PL-Sm:CaF_2_). (a) Schematic representation of a phospholipid (PL) coating of the OA-Sm:CaF_2_ NCs. (b) DLS of Sm:CaF_2_ NCs before (OA-Sm:CaF_2_) and after PL coating (PL-Sm:CaF_2_). (c) *T*_1_ values of PL-Sm:CaF_2_, PL-La:CaF_2_, and PL-CaF_2_ NCs. (d) ^19^F MRI of phantoms composed of PL-CaF_2_, PL-La:CaF_2_, and PL-Sm:CaF_2_ NCs (70 mM ^19^F/sample) dispersed in water, using TR = 4 ms. (e) ^19^F MRI SNR of the studied solutions shown in d. (f) ^19^F MRI of a phantom containing the relevant concentrations of PL-Sm:CaF_2_ (left) and the obtained SNR as a function of NCs’ concentration (right). The ^19^F concentrations in the examined tubes were 5 mM (for 0.9 μM PL-Sm:CaF_2_), 2.5 mM (for 0.45 μM PL-Sm:CaF_2_), and 1.25 mM (for 0.22 μM PL-Sm:CaF_2_). The results shown in panels c–f were obtained with nanofluorides that were not modified with either fluorescent PL (14:0 Liss Rhod PE) or lactosyl PL (18:1 lactosyl-PE).

NCs capped by hydrophobic ligands (such as the OA-CaF_2_ NCs in Figure 1) can be subsequently coated with a monolayer of phospholipids by exploiting the strong hydrophobic interactions between the tails of the capping ligand (OA) and the phospholipid (PL).^6^ Such a procedure (schematically shown in Figure 3a), which endows the NCs with the desired water solubility, can be used to incorporate into these NC targeting ligands and/or fluorescently labeled PLs. In an effort to obtain small, stable, water-soluble PL-Sm:CaF_2_ NCs, we added 1-myristoyl-2-hydroxy-*sn*-glycero-3-phosphocholine (14:0 Lyso:PC), PEGylated PL (18:0 PEG1000-PE), and cholesterol to a solution of OA-Sm:CaF_2_ NCs (relevant amounts are summarized in Table S1). The monodispersity of the resultant PL-Sm:CaF_2_ NCs was maintained in aqueous solution, as confirmed by both TEM images (Figure S8) and DLS measurements, with the expected larger hydrodynamic diameter (11.3 ± 2.8) (Figure 3b) and increased organic-coating mass (Figure S9) compared to the one measured prior to the PL incorporation. PL-Sm:CaF_2_ NCs were found to be stable for at least 1 month in an aqueous buffer, with no observable changes in their size and monodispersity (Figure S10). Importantly, we found the *T*_1_ relaxation time of the PL-Sm:CaF_2_ NCs to be extremely short, 70 ms, more than 2 orders of magnitude shorter (210× shorter) than that obtained for diamagnetically doped PL-La:CaF_2_ NCs (15 s, Figure 3c) in water. This short *T*_1_ value, which was found to be very short at different magnetic fields (Table S2), is comparable to the one obtained for paramagnetic fluorinated nanoemulsions.^46^ Surprisingly, the evaluated *T*_1_ value of the PL-Sm:CaF_2_ NCs in aqueous solution was found to be even shorter than the one calculated for OA-Sm:CaF_2_ dispersed in cyclohexane (Figure S2). This observation might be explained by the different correlation times of the NCs in water, which could be affected by several factors, such their larger size (Figure 3b and Figure S10), the existence of hydration shell only in water, different viscosity of the dispersing solution, hydrogen bonds, or even charge differences.

Next, a phantom composed of three samples (containing similar fluorine concentrations) of non-, diamagnetic-, or paramagnetic-doped nanofluorides (PL-CaF_2_, PL-La:CaF_2_, or PL-Sm:CaF_2_ NCs, respectively, all three are not fluorescent, Figure 3d) was studied in order to evaluate the gain in sensitivity upon *T*_1_ shortening (from 15 s to 70 ms) when using them for ^19^F MRI. The short *T*_1_ of PL-Sm:CaF_2_ NCs (Figure 3c) allows the repetition time of the acquisition to be reduced to only 4 ms with an excitation flip angle of 5° (fulfilling the Ernst angle condition and complying with the hardware limitations) and, thus, a ^19^F MR signal to be obtained from PL-Sm:CaF_2_ NCs within a few seconds (Figure S11). The very short *T*_1_ of PL-Sm:CaF_2_ NCs leads to an 8-fold higher SNR compared to the two types of diamagnetic CaF_2_ NCs (non-doped PL-CaF_2_ and PL-La:CaF_2_) at a given scan time (Figure 3e). Impressively, PL-Sm:CaF_2_ NCs (Figure 3f) yield a SNR of ∼6—at a concentration of only 0.22 μM, which is 1.25 mM of ^19^F content. This represents a more than 40-fold improvement in CaF_2_ detectability over previously proposed PEG-coated CaF_2_ NCs^24^ and a 5-fold improvement over defected CaF_2_ NCs.^37^

### Lactose-Modified CaF_2_ NCs for Immune Targeting

In addition to improved sensitivity (*i.e.*, shorter *T*_1_), robust mapping of ^19^F MRI tracers requires extensive and efficient nanofluoride accumulation at the region-of-interest (ROI) to ensure a detectable number of ^19^F atoms. Therefore, with an eye to enhance ^19^F MRI detectability of inflammation, we synthesized glyconanofluorides by coating Sm:CaF_2_ NCs with lactose moieties to obtain lactose multivalency on the surface of the nanofluoride and to prompt their cellular accumulation (*i.e.*, immunolabeling) within sites of inflammation.^52^ By applying the same synthetic procedure described in Figure 3a, Sm:CaF_2_ NCs were coated with glycosylated phospholipids (18:1 lactosyl-PE). Additionally, fluorescently labeled phospholipids (14:0 Liss Rhod PE) were integrated into the NC’s surface for cellular uptake validation by fluorescence microscopy and fluorescence-activated cell sorting (FACS) analysis. Of note, the intrinsic fluorescent properties of the Sm:CaF_2_ NCs were found to be negligible, with no effect on the fluorescent characteristics of the rhodamine dye (Figure S12). High-resolution mass spectrometry (Figures S13 and S14) confirmed the attachment of the introduced ligands to obtain both lactose-coated, fluorescently labeled nanofluorides (LPL-Sm:CaF_2_) and their nonglycosylated control, PL-Sm:CaF_2_ NCs. Both NC solutions (*i.e.*, LPL-Sm:CaF_2_ and PL-Sm:CaF_2_) showed a similar, small hydrodynamic diameter, were transparent, exhibited the strong characteristic color of the rhodamine dye (Figure S15), and had the identical, typical, high-resolution ^19^F NMR signal of CaF_2_ NCs (−109 ppm, Figure S16). In good agreement with previous reports of increased cellular uptake of lactose-modified, small-sized NCs,^52−54^ incubating a mouse monocyte-derived macrophage cell line (RAW 264.7) with lactose-coated nanofluorides (LPL-Sm:CaF_2_) or nonglycosylated nanofluorides (PL-Sm:CaF_2_) led to a relative increase in the uptake of the former (Figure S17). Furthermore, a toxicity evaluation of the NCs found no evidence of enhanced toxicity by either the Sm^3+^ dopant (3.5 ± 0.5%) or the lactose moiety (Figure S18).

It is important to mention that the *in vitro* toxicity assay does not mimic the situation *in vivo* (due to the dilution of the injected material in the blood, its immediate washout as compared to a lengthy passive incubation and more), and a maximum tolerated dose was evaluated prior to the *in vivo* experiment (and found to be 50 mg/kg body weight). Then, the biodistribution profile of the fluorescent LPL-Sm:CaF_2_ and PL-Sm:CaF_2_ was assessed following their intravenous administration into two respective groups of mice (*N* = 9 for each group). The organs of the mice were excised 30 min (*N* = 3 for each group), 2 h (*N* = 3 for each group), or 24 h (*N* = 3 for each group) after NC administration, and their fluorescent intensity was quantified (Figure S19). No significant difference between the biodistribution profiles and NC clearance could be detected between the LPL-Sm:CaF_2_ and PL-Sm:CaF_2_ groups. A histopathological evaluation of the kidneys of the examined mice showed no evidence of pathological damage 24 h following LPL-Sm:CaF_2_ administration (Figure S20). However, in light of recent concerns regarding Gd^3+^ involvement in nephrogenic systemic fibrosis induction,^55^ additional long-term studies of the effect of the proposed NCs are needed to evaluate their biological and physiological safety prior to considering them for human studies. It is important to note here that, although the obtained fast clearance from all the examined organs without evident damage indicates a very important feature, considering the prolonged half-life of other fluorine-containing contrast agents, this property should be further investigated. Given that the biodistribution evaluation was based on the fluorescent signal of the rhodamine-labeled phospholipid, and in light of previous observations of the high probability of dynamic lipid exchange between nanoparticles and cells’ lipids,^56,57^ future studies to fully characterize nanofluorides’ biodistribution and clearance profiles should likewise be undertaken.

### Glyconanofluorides for Immune Targeting of Inflamed Lymph Nodes *in Vivo*

**Figure 4:**
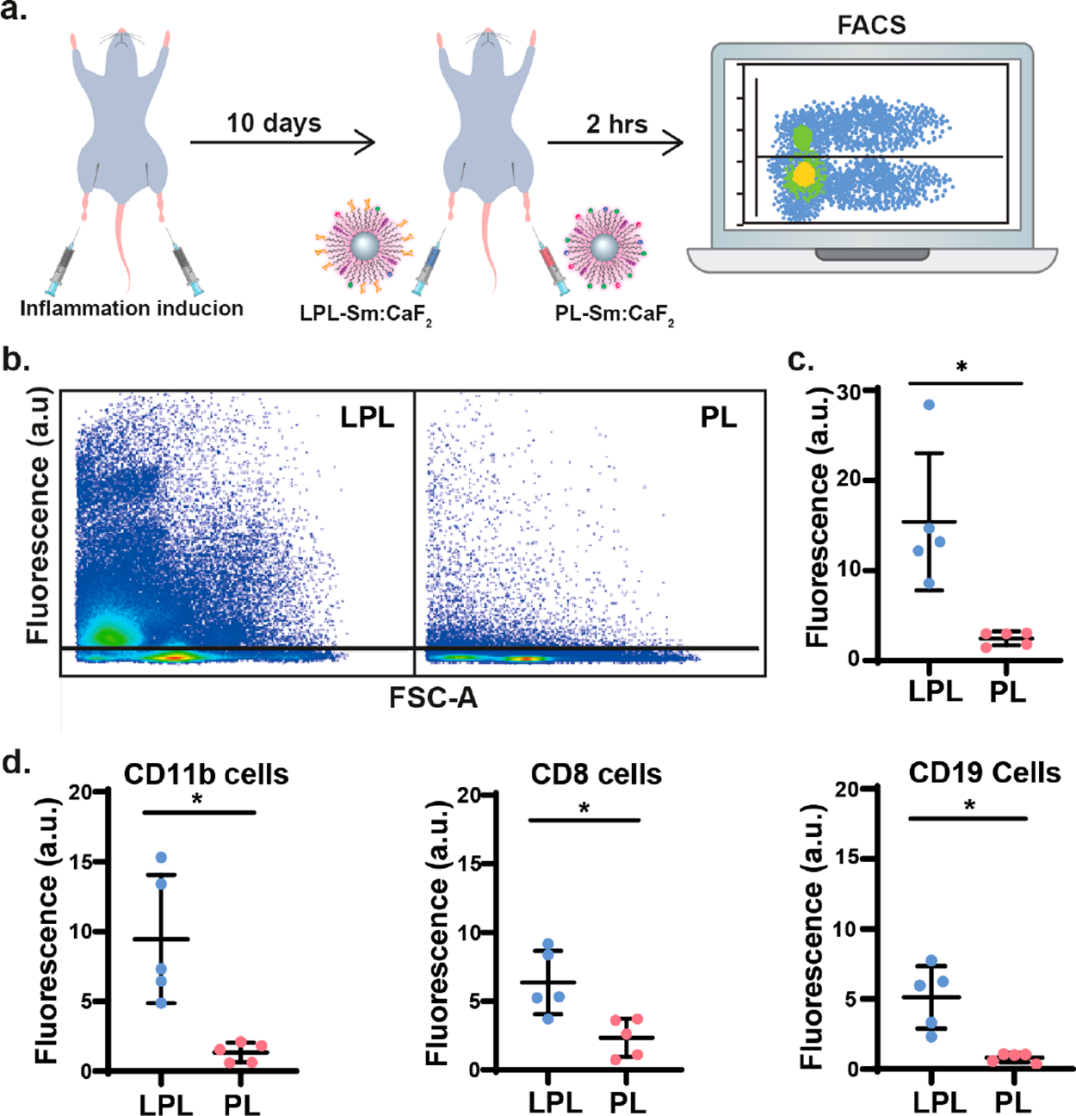
Immune targeting of inflamed lymph nodes *in vivo*. (a) Schematic illustration of the *in vivo* experimental setup used for the injection of both PL-Sm:CaF_2_ (nanofluorides, *i.e.*, PL) or LPL-Sm:CaF_2_ (glyconanofluorides, *i.e.*, LPL) NCs into inflamed mice footpads (20 μL of 25 mg/mL NCs). (b) Representative dot blots of FACS analysis of cells excised from lymph nodes 2 h post-injection of LPL-Sm:CaF_2_ or PL-Sm:CaF_2_ NCs. (c) Quantitative analysis of the FACS data (rhodamine) obtained from five different mice (*N* = 5, Student’s test, * represents a *p* value <0.05). (d) Dot graph representing the lymphatic distribution of LPL-Sm:CaF_2_*vs* PL-Sm:CaF_2_ within subtypes of immune cells that were excised from lymph nodes 2 h post-injection of the NCs (*N* = 5), from left to right: CD11b leukocyte cells, CD8 T-cells, and CD19 B-cells. All studies were performed with fluorescently labeled nanofluorides (either LPL-Sm:CaF_2_ or PL-Sm:CaF_2_).

To assess whether the LPL-Sm:CaF_2_ NCs can serve as background-free imaging tracers for the noninvasive mapping of inflammatory activity, we used an *in vivo* animal model of inflammation. First, the footpads of mice were subcutaneously injected with 50 μL of an immunogenic emulsion, inducing a local inflammation in both right and left hinds. Ten days post-immunization, when extensive inflammatory activity was observed in the lymph nodes (LNs) in the proximity of both immunized hinds, fluorescently labeled lactose-presenting (LPL-Sm:CaF_2_) and control (PL-Sm:CaF_2_) NCs (Figure 4a) were simultaneously injected into the right and left hinds, as noted in the figure. Two hours post-injection, LN cells were harvested and analyzed by FACS (Figure 4b–d). We found the paramagnetic nanofluorides to have no toxic effect on the excised lymphatic cells as compared to the control, even when injected at relatively high concentrations (Figure S21).

Our FACS assessment of the LNs of an inflamed mouse following the nanofluoride injections clearly shows an accumulation of LPL-Sm:CaF_2_ NCs more extensive and much higher than that of PL-Sm:CaF_2_ NCs (Figure 4b), with the quantitative analysis of this data set demonstrating a greater than 6-fold difference between the two studied groups (Figure 4c, *N* = 5). Such a significant accumulation (*p* value = 0.017) of lactose-modified CaF_2_ NCs confirms that glyconanofluorides offer improved immune targeting of inflammatory tissue *in vivo*, even upon their systemic injection. Note here, as summarized in Figure 4d, CD11b leukocytes (7.1-fold higher, *p* value = 0.008, for LPL-Sm:CaF_2_), CD19 B-cells (6.3-fold higher, *p* value = 0.007, for LPL-Sm:CaF_2_), and CD8 T-cells (2.7-fold higher, *p* value = 0.006, for LPL-Sm:CaF_2_) showed significantly higher accumulation of the targeted lactose-coated nanofluorides, representing their lymphatic distribution. These results are in agreement with previous reports that showed that several types of immune cells, including CD11b leukocytes, B-cells, and T-cells, can recognize and bind synthetic carbohydrate formulations.^53,58,59^

### Paramagnetic Glyconanofluorides Allow *in Vivo*^19^F MRI Mapping of Inflammatory Activity

**Figure 5:**
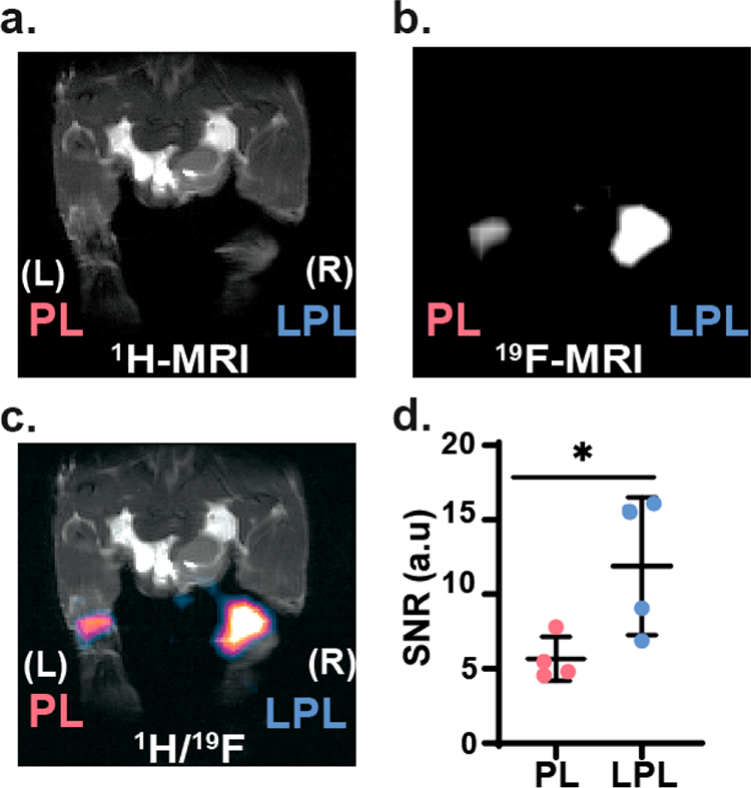
*In vivo*^19^F MRI study of inflamed mice. Ten days post-immunization, mice (*N* = 4) were subcutaneously injected with LPL-Sm:CaF_2_ NCs (right leg, labeled as “R”) and PL-Sm:CaF_2_ NCs (left, labeled as “L”). Then, 2 h post-injection mice were anesthetized and scanned with MRI. (a) ^1^H MRI, (b) ^19^F MRI, and (c) ^1^H/^19^F MRI overlay of a representative mouse. (d) SNR of ^19^F MRI at the LNs ROIs (*N* = 4, Student’s test, * represents a *p* value <0.05). The in-plane resolutions of the ^1^H MR and ^19^F MR images are 0.35 × 0.2 mm^2^ and 1.4 × 0.78 mm^2^, respectively; the slice thickness is 1 mm in ^1^H MRI and 0.78 mm in ^19^F MRI.

To examine the ability to use the obtained glyconanoparticles as nanotracers for background-free *in vivo* mapping of inflammation with ^19^F MRI, we exploited *in situ* labeling of circulating immune cells, an approach frequently used by PFC-based formulations.^27,60^ To do so, 10 days after being immunized, mice (*N* = 4, Figure 5) were subcutaneously injected with 20 μL (25 mg/mL of NCs) of LPL-Sm:CaF_2_ NCs and PL-Sm:CaF_2_ NCs in the right (labeled “R”) and left (labeled “L) footpads, respectively. Two hours later, mice were anesthetized and scanned with both ^1^H MRI (Figure 5a) and ^19^F MRI (Figure 5b). The localization of the detected nanofluoride-derived ^19^F MR signal was confirmed by overlaying the obtained ^19^F MR images on the high-resolution anatomical ^1^H MR images (Figure 5c). In high correlation with the results obtained from the FACS experiments (Figure 4b,c), massive accumulation in the inflamed LN was clearly observed in the leg in which the paramagnetic glyconanofluorides were injected (LPL-Sm:CaF_2_, labeled as “R”) compared to the leg injected with the control nanofluorides (PL-Sm:CaF_2_, labeled as “L”). This significant (*p* value = 0.033) 2-fold higher ^19^F MR signal (Figure 5d) confirms our hypothesis that paramagnetic glyconanofluorides can be used as small (<10 nm), sensitive (*T*_1_ = 70 ms), and specific (lactose-coating) nanotracers for *in vivo* background-free mapping of inflammatory activity with ^19^F MRI.

### Multiplexed *In Vivo*^19^F MRI of Immune Specificity of Glyconanofluorides

**Figure 6:**
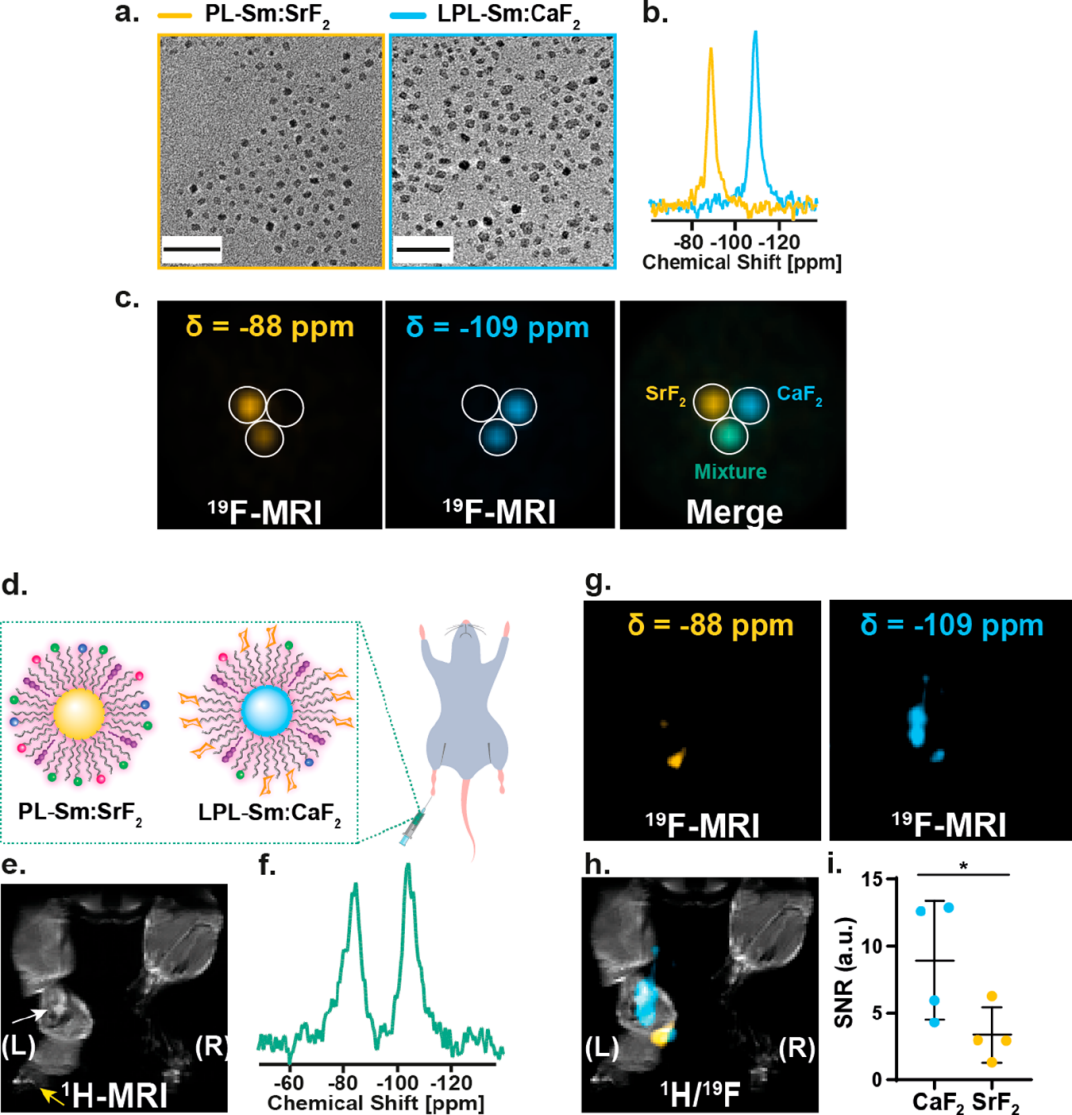
Multicolor immunotargeting with ^19^F MRI. (a) TEM images of PL-Sm:SrF_2_ (yellow frame) and LPL-Sm:CaF_2_ (light blue frame) NCs. Scale bar: 50 nm. (b) ^19^F NMR spectra of PL-Sm:SrF_2_ (yellow) and LPL-Sm:CaF_2_ (light blue) NCs. (c) Multicolor ^19^F MRI of a phantom containing PL-Sm:SrF_2_, LPL-Sm:CaF_2_, or a mixture of both NCs. (d) Schematic representation of nonglycosylated PL-Sm:SrF_2_ and glycosylated LPL-Sm:CaF_2_ NCs injected as a mixture to the footpad of an inflamed mouse. (e) ^1^H MRI of the inflamed mouse; white arrow indicates the inflamed LN, and yellow arrow represents the injection site. (f) *In vivo*^19^F NMR spectrum acquired from the whole volume of the RF coil averaging all of the ^19^F NMR signal of the administrated material (total injected PL-Sm:SrF_2_ and LPL-Sm:CaF_2_). (g) ^19^F MRI acquired with the center of frequency offset set at either −88 ppm (left, yellow) or −109 ppm (right, light blue). (h) Representative ^1^H/^19^F MRI showing the higher accumulation of LPL-Sm:CaF_2_ NCs in the LN. (i) Dot graph presenting the ^19^F MRI signal of either PL-Sm:SrF_2_ or LPL-Sm:CaF_2_ in the LN ROI (*N* = 4, Student’s test, * represents a *p* value <0.05). For the *in vivo* data, the in-plane resolutions of the ^1^H MR and ^19^F MR images are 0.35 × 0.2 and 1.4 × 0.78 mm^2^, respectively; the slice thickness is 1 mm in ^1^H MRI and 0.78 mm in ^19^F MRI.

Capitalizing on the ability to classify different types of synthetic nanofluorides and present them in a “multicolor” fashion, we demonstrated the immune specificity of glyconanofluorides (*i.e.*, LPL-Sm:CaF_2_, colored light blue in Figure 6) over nonglycosylated nanofluorides (*i.e.*, PL-Sm:SrF_2_, colored yellow in Figure 6), in real time, in the same inflamed tissue. This entailed synthesizing Sm^3+^-doped SrF_2_ NCs using the same approach employed for the synthesis of Sm:CaF_2_ NCs. The resultant small-sized, monodispersed Sm:SrF_2_ NCs provided a single high-resolution ^19^F NMR peak that resonated at −88 ppm, as expected,^24^ with the expected short *T*_1_ values (Figure S22). We dispersed Sm:SrF_2_ in water using the synthetic approach shown in Figure 3a, without adding the lactose-modified PLs to obtain PL-Sm:SrF_2_. The nonglycosylated PL-Sm:SrF_2_ was of a similar size and shape to that of the glycosylated LPL-Sm:CaF_2_ (Figure 6a, left) but with well-resolved high-resolution ^19^F NMR peaks that differ from one another by more than ∼20 ppm (Figure 6b), allowing their spatial distribution to be mapped with ^19^F MRI (Figure 6c) and presented in a multicolor manner. The specificity for detecting each of the agents (Sm:SrF_2_ and Sm:CaF_2_) shown in Figure 6c was evaluated by determining the contrast-to-noise ratio at each of the examined frequency offsets (−88 ppm *vs* −109 ppm) for each of the studied particles and showed negligible signals overlapping and thus ultimate specificity (Table S3).

This capability to monitor the two types of nanofluorides (glycosylated *vs* nonglycosylated) simultaneously in the same imaging frame, without overlapping signals and without affecting the two detectable ^19^F MRI signals (Figure 6c, bottom tube, displayed as green color and summarized in Table S3), was used to demonstrate the immune specificity of glyconanofluorides *in vivo*. In this case, an aqueous solution containing similar concentrations of the two types of nanofluorides (PL-Sm:SrF_2_ and LPL-Sm:CaF_2_) was injected into the footpads of inflamed mice (shown schematically in Figure 6d). After the injected mouse was localized in the MRI scanner and a ^1^H MR image acquired to identify the inflamed LN of interest (white arrow in Figure 6e), a ^19^F NMR spectrum was acquired from the whole imaging frame. Importantly, an identical ^19^F NMR signal intensity was obtained (Figure 6f) from the injected PL-Sm:SrF_2_ (−88 ppm) and LPL-Sm:CaF_2_ (−109 ppm) NCs, reflecting their similar concentration in the tissue of the live subject following their administration. Note that, while ^19^F MRI detection is limited to the number of ^19^F spins in the imaging voxel (LPL-Sm:CaF_2_ are expected to accumulate in the LNs in higher levels as compare to PL-Sm:SrF_2_), the ^19^F NMR spectrum averages out the signal of all the spins from the whole imaging volume (similar levels of LPL-Sm:CaF_2_ and PL-Sm:SrF_2_). Therefore, sufficient accumulation of nanofluorides in the inflamed LN is required to reach the detectability level in ^19^F MRI.

Indeed, although the two types nanofluorides were injected as a mixture with equal concentrations (Figure 6f), a much larger accumulation of LPL-Sm:CaF_2_ (light blue in Figure 6g) in the LN was observed, in contrast to a much lower ^19^F MRI signal of PL-Sm:SrF_2_ (yellow in Figure 6g). This ability to map both the targeted and nontargeted nanotracers and present their spatial distribution and targetability in a “multicolor” fashion is demonstrated in Figure 6h, and the 2-fold increase in ^19^F MRI SNR when comparing glyconanofluorides (LPL-Sm:CaF_2_) to nonglycosylated nanofluorides (PL-Sm:SrF_2_) is depicted in Figure 6i. The same 2-fold difference was observed when comparing LPL-Sm:CaF_2_ to PL-Sm:CaF_2_ injected in two different legs and imaged with “unicolor” ^19^F MRI (Figure 5d). This demonstrates the desired capability of spatially mapping multiple nanofabrications, with the added benefit of no unwanted background signal. Further, using simultaneously the same imaging modality, we showcase the potential of using nanofluorides with their targetability and “multicolor” imaging capabilities for studying biological multiplexity in scenarios where simultaneous mapping of multiple targets is required. Note here that the use of an MRI scanner operating at 15.2 T like the one used here is beneficial for “multicolor” MRI studies that yield better spectral resolution in addition to the higher sensitivity obtained at higher *B*_0_. Nevertheless, the preserved short *T*_1_ at lower magnetic fields (Table S2) implies that designed paramagnetic nanofluorides will be applicable also for studies performed with MRI scanners operating at lower, more common magnetic fields.

### Paramagnetic Glyconanofluorides Detect Activated Immune Cells at Sites of Neuroinflammation

**Figure 7:**
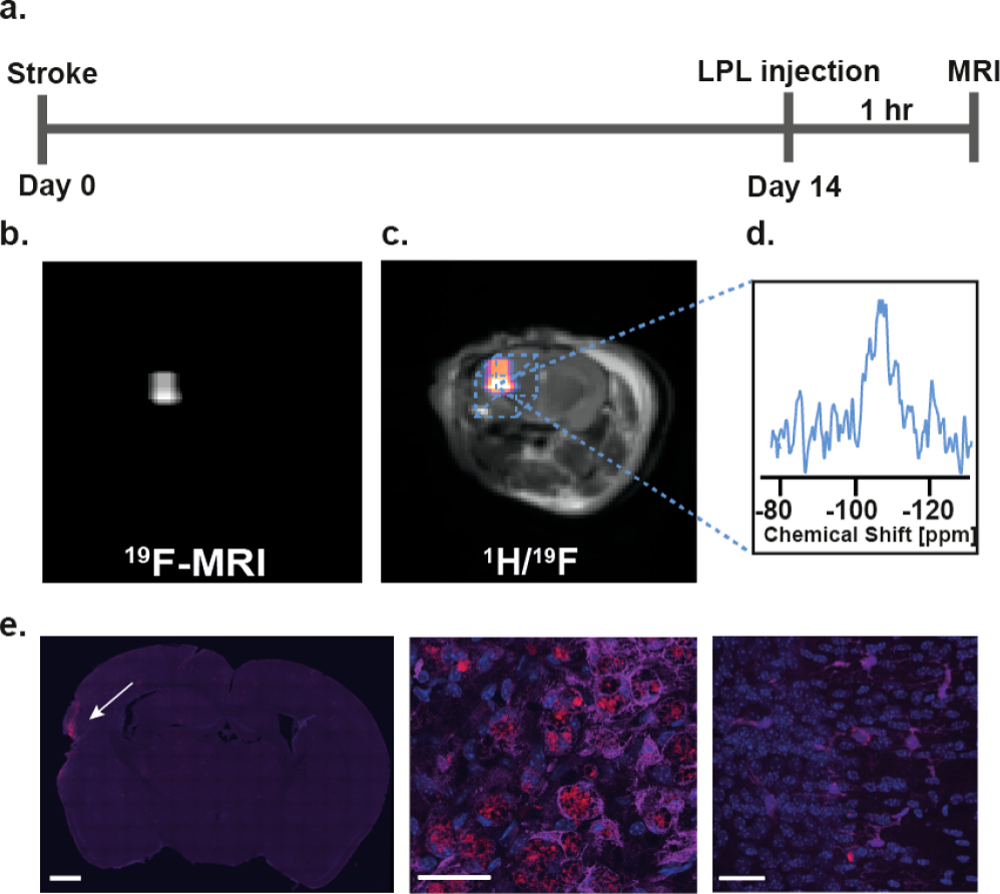
*In vivo* MRI of neuroinflammation. (a) Schematic chart demonstrating the process of stroke induction (day 0) followed by LPL-Sm:CaF_2_ NCs injection and MRI acquisition 2 weeks post-stroke induction. (b) *In vivo*^19^F MRI and (c) ^1^H/^19^F MRI overlay of a representative mouse 14 days after stroke induction (*N* = 4). (d) Localized ^19^F NMR spectrum acquired from the area of the ischemic lesion (marked by a dashed-lined box). (e) Fluorescent microscope images of brain section (20× magnification, left, scale bar: 1 μm) with 63× magnification of the stroke region (middle) and contralateral region (right), stained with DAPI (blue), rhodamine (red, glyconanofluorides), and Iba-1 antibody (magenta). Scale bar: 30 μm. The in-plane resolutions of the ^1^H MR and ^19^F MR images are 0.23 × 0.23 and 0.93 × 0.93 mm^2^, respectively; the slice thickness is 1 mm in both ^1^H MRI and ^19^F MRI.

Finally, we also examined our synthesized glyconanofluorides’ ability to map inflammatory processes within different (nonlymphatic) bioenvironments and pathologies. To this end, we employed glyconanofluorides and ^19^F MRI to visualize the recruitment of activated immune cells to a stroke region in the central nervous system (CNS), which typically peaks between 1^61,62^ and 2 weeks^63−65^ post-ischemic onset. Thus, the injection of the LPL-Sm:CaF_2_ and the ^19^F MRI experiments were performed 2 weeks after the stroke event when the lesion area is expected to be highly necrotic with minimal blood supply and features a massive accumulation of myeloid cells in the lesion and penumbra areas (Figure S23). At this time point after the stroke induction (14 days, Figure 7a), mice were retro-orbitally injected with 20 μL of a LPL-Sm:CaF_2_ (25 mg/mL) solution and then, 1 h later, anesthetized for further ^1^H and ^19^F MRI examination. As hypothesized, the glyconanoparticles accumulated in the lesioned area, as clearly shown for a representative mouse in Figure 7b,c (the examination of all four mice is presented in Figure S24). A localized ^19^F MR spectroscopy of the lesioned region revealed a characteristic ^19^F NMR spectrum of CaF_2_ with the expected −109 ppm resonance (Figure 7d), which further confirmed the accumulation of intact glyconanofluorides in the stroke area. Importantly, using confocal microscopy on brain sections collected from these mice, we observed a massive accumulation of the glyconanofluorides in Iba-1-positive cells recruited to the vicinity of the stroke region (Figure 7e and Figure S25).^66−68^ These observations corroborate our previous results, showing that glycopolymers, such as dextran, accumulate in activated Iba-1-expressing cells 2 weeks after a stroke when their numbers increase around the infarcted area and in the penumbra nearby.^69^ Interestingly, when administered 2 h post-stroke, both fluorescently labeled dextran and LPL-Sm:CaF_2_ were found mostly in the blood vessels and not in Iba-1 expressing cells (Figure S26). Nevertheless, 2 weeks after the stroke, both the glycopolymer (dextran) and the glyconanoparticles (LPL-Sm:CaF_2_) could be found mostly in Iba-1 expressing cells that populated the areas mentioned above (Figure S27). Based on our finding that glyconanofluorides massively accumulate in activated immune cells of inflamed lymph nodes (Figures 4**–**6), the results shown in Figure 7 and Figures S23–S27 strengthen the conclusion that the ^19^F MRI signal obtained at the stroke region is a result of neuroinflammation activity.

## Conclusion

Small-sized glyconanoparticles imitate natural glycan-based structures of pathogens for enhanced immune targeting and can alter their properties (*i.e.*, multivalency, size, charge, density, *etc.*) for a desired purpose.^70^ The paramagnetic nanofluorides introduced here, which exhibit enhanced ^19^F MRI sensitivity, offer a small-sized inorganic NC-based platform for the synthesis of nanosized glycomimetics. Using the paramagnetic dopant Sm^3+^, which induces a significant PRE effect for shortening the *T*_1_ of nanofluorides by more than 200-fold without affecting their already short *T*_2_ values, an 8-fold enhancement in their ^19^F MRI SNR was obtained. By modifying the surface of the paramagnetic nanofluorides with multivalent lactose moieties, we obtained paramagnetic glyconanofluorides that could detect activated immune cells at sites of inflammation following their systemic administration, which we were able to present as hotspot MRI maps. The proposed approach for immune targeting and spatial *in vivo* mapping of inflammatory activity, demonstrated here in both footpad-induced inflammation and in neuroinflammation following ischemic stroke models, could be extended to other diseases in which inflammation plays key roles.^71,72^ In light of the recent advances in engineering small-sized (∼10 nm), nature-inspired mimetics,^14^ we envision that the proposed paramagnetic nanofluorides could be further evolved to yield a wider range of designs and applications.

## Methods

### Synthesis of Oleate-Coated CaF_2_/Doped-CaF_2_/Doped-SrF_2_ NCs

In a typical synthesis, 4.2 mL of oleic acid, 12 mL of ethanol, and 0.1 g of sodium hydroxide were mixed under vigorous stirring in a round-bottom flask at room temperature (RT) for 6 h. To the resultant homogeneous milky mixture were added at once 5 mL of an aqueous solution containing 2 mmol M(NO_3_)_2_ × 4H_2_O (M = Ca^2+^, Sr^2+^), the relevant mol % of Ln^3+^ precursor (added as Ln(NO_3_)_3_ salt), and 5 mL of a 4 mmol NaF aqueous solution. The obtained mixture was vigorously stirred for 1 h and then heated to 160 °C in a Teflon-lined sealed autoclave reactor for 16 h. The reaction mixture was left to cool at RT and then centrifuged at 8500 rpm for 10 min. The obtained precipitate was washed three times with cyclohexane/ethanol (1:10 ratio), followed by 10 min centrifugation at 6500 rpm. The final product was obtained as a white solid, which was redispersed in 2 mL of cyclohexane, followed by an additional centrifugation for 3 min at 2000 rpm in order to remove any remaining impurities and aggregates. The resultant clear solution was kept in a glass vial for further characterization.

### Fabrication of Glyconanofluorides

In order to provide the suspended hydrophobic lanthanide-doped NCs (either Ln:CaF_2_ or Ln:SrF_2_) with the required water solubility for biological applications, their surface was covered with phospholipids, which contain both hydrophobic and hydrophilic regions. In a typical synthesis, 30 mg of myristoyl hydroxyphosphatidylcholine, 2 mg of cholesterol, 5 mg of 18:0 PEG1000-PE, and 0.15 mg of 18:1 lactosyl-PE (for the lactose-modified NCs) were dissolved in 5 mL of a 20:1 chloroform/methanol solvent mixture. This solution was then added to 2 mL of chloroform containing 30 mg of the dissolved desired NCs. The resultant lipid-coated NC solution was then added dropwise to 30 mL of deionized water at 80 °C under vigorous stirring and left to cool to RT. Next, it was centrifuged at 2000 rpm for 3 min, and the sediment (large aggregates or unsuspended nanoparticles) was discarded. To concentrate the supernatant, it was transferred to a centrifugal filter unit (MWCO 10 kDa) and centrifuged at 5000*g* for several 15 min cycles until the total volume reached 500 μL. Then, the sample went through several dialysis cycles, either in ultrapurified water, to remove excess unbound phospholipids, or in phosphate buffer saline (PBS), for further biological usage.

### Animal Studies

All animal studies were performed in accordance with the Weizmann Institute’s Institutional Animal Care and Use Committee (IACUC) guidelines and regulations.

#### Local Inflammation Induction

Eight-week-old female SJL/J mice were immunized by the subcutaneous injection of 50 μL of an immunogenic emulsion (in PBS) composed of complete Freund’s adjuvant containing 150 μg of *Mycobacterium tuberculosis* H37Ra. Mice were then placed in their cage for 10 days to develop inflammation.

#### Stroke Induction

This stroke model was previously described by Karatas *et al.*(73) Briefly, mice were anesthetized with 4% isoflurane in an induction chamber, placed in a stereotaxic frame, and kept on 1% isoflurane. The body temperature was maintained at 37 ± 0.1 °C using a homeothermic blanket control unit (TC-1000, CWE INC, USA). Surgery was performed under an operating biomicroscope. The scalp was opened, and the cranial sutures and bregma were exposed. The right temporal muscle was pushed aside until the squamous part of the temporal bone was exposed. The area just above the junction between the zygomatic arch and the squamous bone was thinned, using a high-speed drill, and cooled with saline. The trace of MCA was visualized through the thinned temporal bone. The thinned bone was removed carefully to avoid damaging the MCA. A piece of 30% FeCl_3_-saturated filter paper (0.3 × 1 mm^2^) was placed over the intact dura along the trace of the MCA, starting from the M1 branch. After 20 min, the FeCl_3_-saturated filter paper was removed and a clot was observed in the MCA, under the operating biomicroscope. The right temporal muscle was placed back, and the scalp was closed using Vetabond 3M (3M, Minneapolis, USA). Mice were then placed in a preheated cage for recovery and allowed free access to food and water.

#### Fluorescence-Activated Cell Sorting

All FACS experiments were performed 10 days post-immunization when maximum inflammatory activity is expected in the above-mentioned animal model. Mice were sacrificed 2 h post-nanofluoride injection (20 μL of 25 mg/mL of NCs), and the cells of their popliteal LNs were harvested and suspended in PBS for FACS analysis using a LSR II flow cytometer (BD Biosciences). Quantitative analysis of fluorescent cells was performed using Flowjo software (version 10, TreeStar, Oregon, USA).

#### Toxicity Determination of Paramagnetic Nanofluorides

Lymphatic cells were harvested at day 10 post-immunization from two groups of mice (*N* = 6 mice in each group) following the subcutaneous injection (20 μL of 75 mg/mL of NCs) of either LPL-Sm:CaF_2_, PL-Sm:CaF_2_, or PBS (control). The viability of nonfixed lymphatic cells was evaluated by a commonly used DNA-binding dye assay using 4′,6-diamidino-2-phenylindole (DAPI). Excised cells were resuspended in PBS and stained for 20 min at 4 °C with 1 μg/mL DAPI solution. Cells were then washed twice with cold PBS and immediately analyzed by flow cytometry using a UV laser (355/450 nm).

#### Evaluation of Lymphatic Accumulation of Nanofluorides

Ten days post-immunization, five mice were simultaneously injected with 20 μL of glycan-presenting (LPL-Sm:CaF_2_) and control (PL-Sm:CaF_2_) NCs in their right and left hind, respectively. Two hours post-injection, mice were sacrificed and popliteal lymph node cells were immediately harvested and suspended in PBS for FACS analysis of the fluorescence of NC accumulation.

### Magnetic Resonance Imaging

All MRI experiments were performed on a 15.2 T horizontal scanner (Biospec, Bruker) using a dual resonator ^1^H/^19^F 23 mm volume coil.

For ^19^F imaging, standard UTE-3D sequence (provided by Bruker) was used, using the following parameters: dummy scans = 250, duration = 1000 ms, bandwidth = 100,000 Hz, excitation-pulse length = 0.0043 ms, excitation pulse bandwidth = 300,000 Hz, receiver gain = 203.

#### Phantom Experiments (*in Vitro*)

In order to evaluate the gain in SNR upon the doping of CaF_2_ NCs (5 mol % of dopant), water-soluble nanofluorides (PL-CaF_2_, PL-La:CaF_2_, and PL-Sm:CaF_2_) were placed in 5 mm NMR tubes at a final fluoride concentration of 70 mM for each of the studied samples. First, ^1^H MRI was acquired using the RARE (rapid acquisition with relaxation enhancement) sequence to obtain the localization of the tubes with the following parameters: RARE factor = 8, TR/TE = 1000/20 ms, 1 mm thick slice, FOV = 2.0 × 2.0 cm^2^, matrix size = 128 × 128, spatial resolution = 0.015 × 0.015 cm^2^ with 1 average and experiment time of 16 s. Then, ^19^F MRI was acquired using a three-dimensional ultrashort TE (3D-UTE) acquisition scheme with the following parameters. For Figure 2c, TR = 4 ms, TE = 8 μs, FOV = 2.0 × 2.0 × 3.0 cm^3^, matrix size = 32 × 32 × 32, spatial resolution = 0.062 × 0.062 × 0.093 cm^3^. The ^19^F MRI acquisition was completed in 25 min using 128 number of averages. For Figure 2e, TR = 4 ms, TE = 8 μs, FOV = 3.2 × 3.2 × 3.2 cm^3^, matrix size = 32 × 32 × 32, spatial resolution = 0.1 × 0.1 × 0.1 cm^3^. The signal was an average of 128 times, and the ^19^F MRI data were acquired in 1 h.

#### *In Vivo* MRI of Inflamed Mice

In order to prevent any residual ^19^F MR signal of fluorinated anesthetics (*i.e.*, isoflurane) that may cause a ^19^F MRI background signal, mice were anesthetized by an intraperitoneal injection of 1 mg/kg medetomidine (Dormitor) and 75 mg/kg ketamine. Immunized mice (*N* = 4) were injected subcutaneously with 20 μL (25 mg/mL) of LPL-Sm:CaF_2_ NCs and PL-Sm:CaF_2_ NCs in the right and left footpad, respectively. Two hours after the nanofluoride injections, the mice were placed on in the MRI scanner, and ^1^H MRI was acquired to obtain high-resolution anatomical images of the scanned mice with both RARE and 3D-UTE protocols. ^1^H RARE was acquired with the following parameters: RARE factor = 8, TR/TE = 1000/5 ms, 19 slices of 1 mm thickness, FOV = 4.5 × 2.5 cm^2^, matrix size = 128 × 128, spatial resolution = 0.035 × 0.019 cm^2^ with 1 average and an experiment time of 16 s. ^1^H 3D-UTE was acquired with the following parameters: TR = 4 ms, TE = 8 μs, FOV = 4.5 × 2.5 × 2.5 cm^3^, matrix size = 128 × 128 × 128, spatial resolution = 0.035 × 0.019 × 0.019 cm^3^ with 1 average that resulted in a scan time of 3 min. Then, ^19^F MRI was acquired using a 3D-UTE sequence with TR = 4 ms, TE = 8 μs, matrix size = 32 × 32 × 32, and 150 averages resulted in a scan time of 30 min. It is important to mention that the FOV dimensions were adjusted to those used to acquire the ^1^H MR image to allow an accurate overlay of the ^19^F MR images on the anatomical ^1^H MR images.

#### *In Vivo* Multiplexed MRI

Immunized mice were injected subcutaneously with 20 μL of a mixture of Lac-PL-Sm:CaF_2_ NCs and PL-Sm:SrF_2_ NCs (25 mg/mL). Mice were then placed on the MRI scanner, and ^1^H MRI was acquired to obtain high-resolution anatomical images of the scanned mice with the RARE protocol. ^1^H RARE was acquired with the following parameters: RARE factor = 8, TR/TE = 1000/20 ms, 15 slices of 1 mm thickness, FOV = 4.5 × 2.5 cm^2^, matrix size = 128 × 128, spatial resolution = 0.035 × 0.019 cm^2^ with 1 average and an experiment time of 16 s. Then, ^19^F MRI was acquired using a 3D-UTE sequence with TR = 4 ms, TE = 8 μs, matrix size = 32 × 32 × 32 cm^3^, spatial resolution = 0.14 × 0.078 × 0.14 cm^3^, and 100 averages, resulting in a scan time of 20 min. The ^19^F signals were acquired separately, once with the RF excitation pulse set to the frequency of CaF_2_ (δ=-109 ppm), bandwidth set to 16,000 Hz, based on the width of the ^19^F NMR signal at the baseline, derived from its ^19^F spectrum, and once for the SrF_2_ frequency (δ = −88 ppm) with the relevant bandwidth. It is important to mention that the FOV dimensions were adjusted to those used to acquire ^1^H MRI to allow further accurate overlay of the ^19^F MR images on the anatomical ^1^H MR images. The ^19^F NMR spectrum of the injected mice was acquired using a simple ^19^F single-pulse protocol, with the following parameters: TR = 200 ms, TE = 50 μs with 500 scans, which resulted in a 2 min scan time.

#### *In Vivo* MRI of Mice after Stroke

Fourteen days after stroke, mice (*N* = 4) were injected retro-orbitally with 20 μL (25 mg/mL) of LPL-Sm:CaF_2_ NCs. One hour after the nanofluorides injection, the mice were placed in the MRI scanner and ^1^H MRI was acquired to obtain high-resolution anatomical images of the scanned mice with both RARE and 3D-UTE protocols. ^1^H RARE was acquired with the following parameters: RARE factor = 8, TR/TE = 1000/5 ms, 19 slices of 1 mm thickness, FOV = 4.5 × 2.5 cm^2^, matrix size = 128 × 128, spatial resolution = 0.035 × 0.019 cm^2^ with 1 average and an experiment time of 16 s. ^19^F MRI was acquired using a 3D-UTE sequence with TR = 4 ms, TE = 8 μs, FOV = 3.0 × 3.0 × 3.2 cm^3^, matrix size = 32 × 32 × 32, and 150 averages resulted in a scan time of 30 min.

All other experimental procedures, characterization data, and supporting figures are provided in the Supporting Information.

## Supporting Information Available

Additional descriptions of the performed experiments and supporting figures (PDF)
