# Supplementary material for: Glyconanofluorides as Immunotracers with a Tunable Core Composition for Sensitive Hotspot Magnetic Resonance Imaging of Inflammatory Activity
Source: ACS Nano. 2021 Apr 19;15(4):7563–74. doi: 10.1021/acsnano.1c01040 (PMC8155386; doi:10.1021/acsnano.1c01040)
Supplement: Supplementary file 1 — nn1c01040_si_001.pdf [file nn1c01040_si_001.pdf]

Supporting Information

Glyconanofluorides as Immunotracers with a Tunable Core Composition for Sensitive Hotspot Magnetic Resonance Imaging of Inflammatory Activity

Dana Cohen[1], Reut Mashiach[1], Lothar Houben[2], Andrea Galisova[1], Yoseph Addadi[3], David Kain[4], Alisa Lubart[4], Pablo Blinder[4], Hyla Allouche-Arnon[1], Amnon Bar-Shir[1*]

[1]Department of Organic Chemistry, Weizmann Institute of Science, Rehovot 7610001, Israel.

[2]Department of Chemical Research Support, Weizmann Institute of Science, Rehovot 7610001, Israel. [3]Life Sciences Core Facilities, Weizmann Institute of Science, Rehovot 7610001, Israel.

[4]Neurobiology, Biochemistry and Biophysics School, George S. Wise Faculty of Life Sciences, Tel Aviv University, Tel Aviv 69978, Israel.

*Corresponding Author: amnon.barshir@weizmann.ac.il

Keywords: Glycomimetic, Immune-targeting, Inflammation, Glyconanoparticles, ^{19}F-MRI, multicolor MRI

Supporting Methods……………………………………………………………Pages 2-7
Supporting Figures…………………………………………………………..Pages 8-20
Supporting Tables……………………………………………………………Pages 21-22

Supporting Methods

Centrifuge

All centrifugation procedures were performed using a 5810 R Eppendorf centrifuge with a F-45-6-30 fixed angle rotor, allowing centrifugation of a sample at a maximum of 12,000 rpm.

Dynamic Light Scattering (DLS)

The average hydrodynamic diameter and size distribution of the obtained NCs in solution were evaluated by DLS with a Malvern Nano-ZS. Measurements were performed in a 12.5 mm diameter cylindrical quartz cuvette for NCPs in organic solvents (oleate-coated NCs) and in a polystyrene disposable cuvette for NPs in aqueous solution (water-soluble NCs).

Electron Microscopy

Sample preparation: (*i*) For oleate-coated NPs, one drop of the suspension (NCs in cyclohexane) was placed on a CF300-Cu grid followed by soaking the solvent with kimwipe paper for better spread. Then, the grid was left to dry for several hours prior to the measurements. (*ii*) For water-soluble NCs, the sample was prepared using the same procedure with a plasma treatment (one minute) prior to the adsorption of the NCs solution, for better adherence. Then, the grid was left to dry for at least 12 h before starting the analysis.

(i) Transmission electron microscope (TEM): The size and shape of the NCs were determined by TEM and high-resolution TEM (HRTEM). The TEM data were acquired with a JEOL JEM 2100 LaB_6 microscope at an accelerating voltage of 200 kV. Images were recorded with a Gatan Ultrascan 1000 XP CCD camera. High-resolution TEM images were collected on the Themis-Z (Thermo Fisher Scientific Microscopy Solutions, Hillsboro, USA) instrument at 80 kV acceleration voltage on a Gatan OneView CMOS camera (Gatan Inc., Pleasanton, USA) at negative spherical aberration settings (Cs=-20 um) with higher-order axial aberrations corrected up to 30 mrad.

(ii) Scanning Transmission Electron Microscope (STEM): STEM images and EDS hyperspectral maps were recorded in a probe aberration-corrected FEI Titan ChemiSTEM (Thermo Fisher Scientific Microscopy Solutions, Hillsboro, USA) and a double aberration-corrected Themis-Z transmission electron microscope equipped with a Schottky type high-brightness electron gun and CEOS double hexapole aberration correctors at an acceleration voltage of 200 kV and 80 kV, respectively. High-resolution HAADF images were recorded with a semi-convergence angle of 30 mrad and a probe current of 50 pA on a Fischione Model 3000 detector with a collection angle of 80-300 mrad, and bright field images on an on-axis FEI BF detector with a collection angle

of 43 mrad. EDS hyperspectral data were obtained with a semi-convergence angle of 30 mrad at a beam current of 200 pA on a windowless Super-X EDS detector.

Thermogravimetric Analysis (TGA)

The relative decomposition of the organic ligand on the synthetic NCs was determined by thermo gravimetric analysis (TGA). Prior to the analysis, purified NCs were dried using a high-vacuum pump to remove any residual organic solvents and water. 5 mg of dry nanoparticles were analyzed with an SDT Q 600 (TA Instruments) instrument. The measurements were performed under an N_2 atmosphere using an Alumina (Al_2O_3) sample pan at a temperature ranging from ambient to 1000°C, with a heating rate of 20 °C/min.

High-resolution electrospray ionization mass spectrometry (ESI-Q-Tof-MS)

The analyses were carried out on a Waters Xevo G2-XS QTof mass spectrometer (Manchester, UK) with an electrospray ionisation (ESI) source operating in negative mode. The solutions were directly infused at a flow rate of 10µL/min. All spectra were acquired in the mass range of 50–2000 m/z. The mass errors of the analyzed spectra are no more than 5.0 ppm. Analyses were performed using a capillary voltage of 2.20 kV, cone gas flow was 28 L/hr, source temperature was set at 120°C and cone voltage of 40V. The desolvation temperature was set at 250°C and the desolvation gas (N2) flow rate was set at 400 L/hr. All measurements were done using Leucine-Enkephalin (200µg/uL, acetonitrile:H_2O containing 0.1% formic acid (1:1, v/v)) as a lockspray reference at flow rate of 10uL/min to ensure mass accuracy and follow resolution mode. Data acquisition and recording was done by Waters MassLynx v4.2 software. Synthetic Lactosyl PE (860080, Avanti, Polar Lipids, Inc. US) was also measured as analyze.

Matrix-Assisted Laser Desorption Ionization Time-of-Flight Mass Spectrometry (MALDI TOF/TOF MS)

3µL of each tested sample solution were mixed well with the same volume of a matrix solution comprising a saturated solution of α-Cyano-4-hydroxycinnamic acid (CHCA, Sigma-Aldrich) dissolved in methanol. Then, 0.5µL of this mixture was spotted three times on a MALDI target plate (Opti-TOF 384-Well Insert, AB Sciex, Framingham, MA, USA). The mixture was then left to dry at room temperature. Each spot was measured three times. MALDI-MS spectra were recorded on an AB Sciex TOF/TOF 5800 mass spectrometer instrument (Applied Biosystems inc., Foster City, CA, USA). The spectra were recorded in the reflectron negative ion mode within a mass range of m/z 500 to 5000, with a focus mass of 2750 Da. The following parameters were set

as follows: bin size of 4 ns, final detector voltage of 1.500 kV with a multiplier value of 0.50, laser intensity of 5535. The total ion chromatograms were acquired and analyzed using the Data Explorer 4.11 software (AB SCIEX, Framingham, MA, USA).

High-resolution Nuclear Magnetic Resonance (NMR)

Freshly synthesized and purified nanofluorides (either oleate-coated or water-soluble) were studied with high-resolution ^{1}H and ^{19}F-NMR. PL-coated nanofluorides were also studied with ^{31}P-NMR. NMR experiments were performed on either 7.0 T or 9.4 T NMR spectrometers (Bruker, Germany), with the sample temperature stabilized at 298°K. ^{1}H-NMR spectra (300 MHz for 7.0 T and 400 MHz for 9.4 T) were acquired for all samples prior to the ^{19}F-NMR experiments. High-resolution ^{19}F-NMR (282.2 MHz for 7.0 T and 376.7 MHz for 9.4 T) spectra were acquired for all samples, followed by longitudinal (T_1) and transverse (T_2) relaxation-time evaluations. T_1 and T_2 relaxation times were calculated using inversion recovery (IR) and Car-Purcell-Meiboom-Gill (CPMG) experiments, respectively.

Cell uptake assay

RAW 267.4 cells were incubated 24 hr before the assay (30k). Lac-Fl-PL-Sm:CaF_2 and Fl-PL-Sm:CaF_2 NCs were incubated with the cells for 24 hr , then washed out and imaged under the microscope.

CCK-8 Assay for cell toxicity study

RAW264 cells (12×10^3 cells/well) were cultured in a 96-well microplate with Dulbecco Modification of Eagle medium (DMEM, High Glucose) for 24 h at 37 °C and 5% CO_2. Then, cells were treated with water-soluble either PL-Sm:CaF_2 / LPL-Sm:CaF_2 / LPL-CaF_2 NCs over a range of concentrations. After 1.5 hrs of incubation, the incubating medium was replaced with 100 µl fresh medium and followed by the addition of WST-8 solution (10 µl) to each well for additional 3 hrs of incubation at 37°C. The absorbance at 460 nm was measured using a plate reader. Cell viability was calculated as a percentage of the treated cells from that of the control (non-treated living cells). For each concentration of NCs, the average absorbance value was calculated from 8 biological replicates. Cells treated with 50% DMSO were used as positive control to demonstrate the response of the sassy to dead cells

Biodistribution study

Seven-weeks old CB57BL male mice were retro-orbitally injected with 70 µL of either PL-Sm:CaF_2 or LPL-Sm:CaF_2 solution. The control animal received the same volume of PBS. At 30 min, 2 hrs or 24 hrs after injection, the mice were anesthetized and intracardially perfused with PBS (N = 3 per each time point). The brain, liver, heart, kidneys, spleen, intestine and lungs were removed and fixed overnight in 4% formaldehyde, then the organs were washed two times and placed in PBS. The fluorescence images of fixed organs were acquired on a Spectrum IVIS optical imager (Perkin Elmer, USA) using the following parameters: exposure time of 10 s (1 s for intestines), aperture 4, excitation at 535 and emission at 600 nm (according to Rhodamine conditions). The fluorescence signal was measured from the region of interest covering the whole organ and expressed as average radiant efficiency ([photons/s/cm^2/sr]/(µW/cm^2) using the Living Image software (Perkin Elmer, USA). Each organ was normalized to its control (injected with PBS).

Lymphatic distribution FACS analysis

Ten days post immunization, Rhodamine labeled of either PL-Sm:CaF_2 NCs or LPL-Sm:CaF_2 NCs, were subcutaneously injected to the foot pads. Two hours post injection, mice were sacrificed and popliteal lymph nodes cells were immediately harvested and suspended in PBS for FACS analysis. Suspended cells were stained with flurochrome labeled monoclonal antibodies for surface antigen detection. APC conjugated anti mouse CD8, FITC conjugated anti mouse CD19, and FITC conjugated anti mouse CD11b were purchased from BioLegend and used according to the manufacturer's protocols. All FACS studies were acquired using LSRII (BD Biosciences) cell analyzer flow cytometers running FACSDiva software, and all data were processed by using FlowJo analytical software (Tree Star).

Dextran injection and imaging

Fluorescently-labeled Dextran (70kDa) in PBS was retro-orbitally injected either 2 hours or two weeks following stroke induction. Following dextran administration, mice were anesthetized (75 mg/kg Ketamine, 1 mg/kg Dexmedetomidine in 0.9% saline) and perfused with 4% PFA in PBS solution (wt/vol). Mice brain were carefully harvested and fixed with 2.5 % PFA for 24 hours, followed by their submersion in 30% (wt/vol) sucrose solution (in PBS) for 24–48 h in order to avoid structural changes following freezing process of the brains. Brains were washed with PBS to remove sucrose solution excess and immediately froze on dry ice for cryopreservation. Brains were

transferred to a precooled (with dry ice) cryotome and cut to 30-μm-thick slices. Finally, slices from the regions of interest were mount on microscope slides and imaged using an inverted Leica DMI8 wide field fluorescent microscope and confocal microscope (as described above). All fluorescent images were analyzed and processed using FIJI software.

Confocal imaging of brain slices

Fixed brain slices were imaged by Spinning disc confocal system - Dragonfly (Andor Technology PLC) connected to a Leica Dmi 8 microscope (Leica GMBH). Slide overview was performed with 20 X / 0.75 air objective, whole brain slice was imaged by 175 tiles each , with FOV = 665 × 665 μm^2 with 10% overlap and stitched together.

Imaging conditions:

- 1μm slice interval
- 3 Channels;

I. Nuclear – DAPI staining; Ex 405nm, Em 450/50nm

II. Lac-Sm:CaF_2 labeled with Rhodamine; Ex 561nm, Em 620/60nm

III. Iba-1 staining; Ex 637nm, Em 700/75nm

- Laser power for all lasers set to 20%
- Signal was detected by a sCMOS Zyla (Andor) 2048 × 2048, 16 bit
- Camera set for 100ms exposure time
- Disc pinhole – 40 μm
- Lens 20 X / 0.75 air imaging and FOV = 665 × 665 μm^2 , pixel size = 0.324 μm

High magnification z stack of specific location was imaged using the same channels conditions, with a 63X / 1.2 water objective, FOV = 211 × 211 μm^2 (XY), pixel size = 0.103 μm. Total Z of 40 , 21.4 μm slices (step size 0.535 μm).

Histopathological and immunohistochemical staining analyses

The kidneys (excised as described above for the biodistribution evaluation experiments) were snap frozen using dry ice and placed into -80 °C until further processing. Then the organs were placed into 1% formaldehyde solution with PBS for 24 hours before staining. PFA fixed kidney tissues were mounted on paraffin blocks. The blocks were sectioned and tissue slices were heated on a hot plate to remove paraffin. The sections were immersed into a fresh solution of xylene to completely remove the remaining paraffin. The sections were rehydrated followed by hematoxylin staining of the cell nuclei. The dye was removed from the specimens with warm water and were rinsed using ddH2O followed by their immersion in 95% ethanol. The ethanol-treated specimens were counter-

stained with eosin for 1min. Specimens were dehydrated with 70%, 95% and 100% ethanol, followed by a dehydration step in xylene. The final dehydrated kidney specimens were mounted on glass slides with 50% glycerol and observed under a light microscope equipped with a digital camera. Detailed steps of dehydration and immunostaining procedures of the kidney tissues are described in Table S4 and S5, respectively.

Supporting Figures

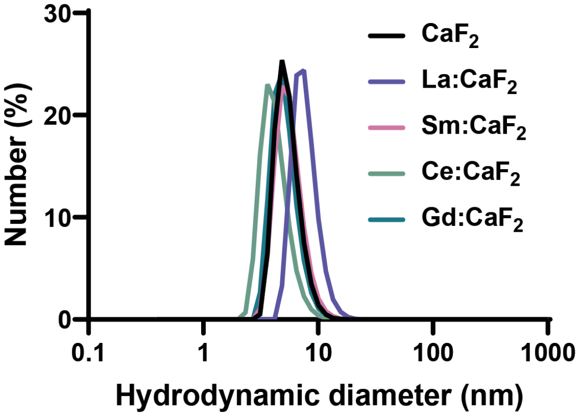

Figure S1. DLS measurements. Size distribution, by number, of CaF_2 (non-doped NCs, black), La:CaF_2 (diamagnetic-doped NCs, purple) , Sm:CaF_2 (paramagnetic-doped NCs, pink), Ce:CaF_2 (paramagnetic-doped NCs, green) and Gd:CaF_2 (paramagnetic-doped NCs, turquoise).

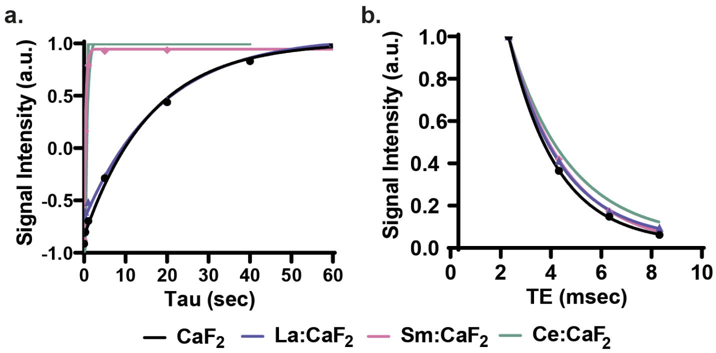

c.

	T_1 (s)	T_2 (ms)	T_1/T_2
CaF_2	16.34 ± 0.03	2.1 ± 0.008	7,781
La:CaF_2	17.56 ± 0.06	2.3 ± 0.01	7,634
Sm:CaF_2	0.33 ± 0.04	2.2 ± 0.002	150
Ce:CaF_2	0.35 ± 0.01	2.7 ± 0.02	130

Figure S2. Relaxation measurements of OA-CaF_2 and OA-Ln:CaF_2 dispersed in cyclohexane. Experimental plots of solutions containing OA-CaF_2, OA-La:CaF_2, OA-Sm:CaF_2 and OA-Ce:CaF_2 that were obtained and used to evaluate the T_1 and the T_2 relaxation times of the fluoride content in nanofluorides from a. Inversion Recovery (IR) and b. Carr-Purcell-Meiboom-Gill (CPMG) experiments, respectively. c. Table summarizing T_1 and T_2 values that were calculated from either IR or CPMG, respectively.

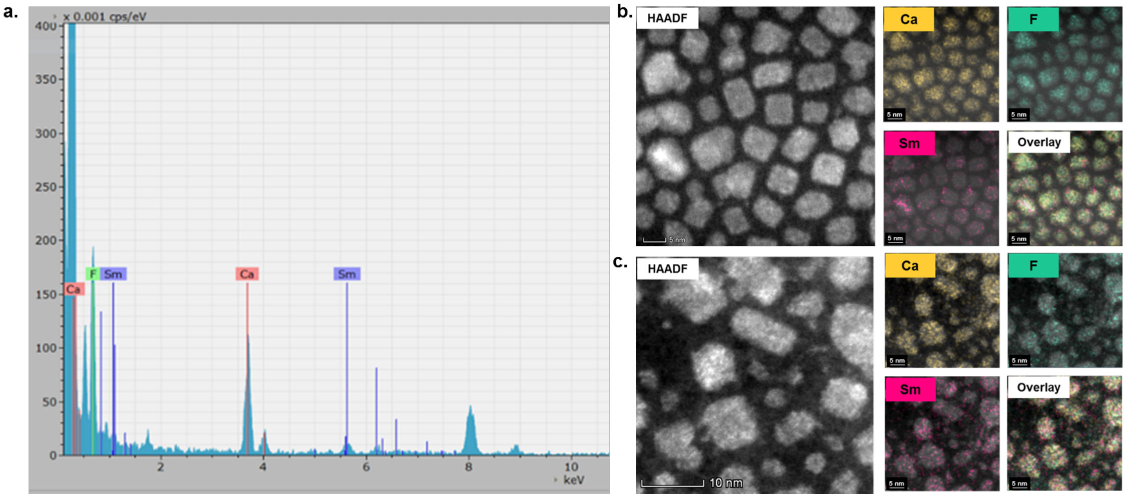

Figure S3. Electron microscopy analysis of OA-Sm:CaF_2 NCs. a. EDS spectrum as evidence for the presence of Sm elements in the fabricated CaF_2 NCs. b. Elemental maps of 10%Sm and c . of 20%Sm.CaF_2 NCs.

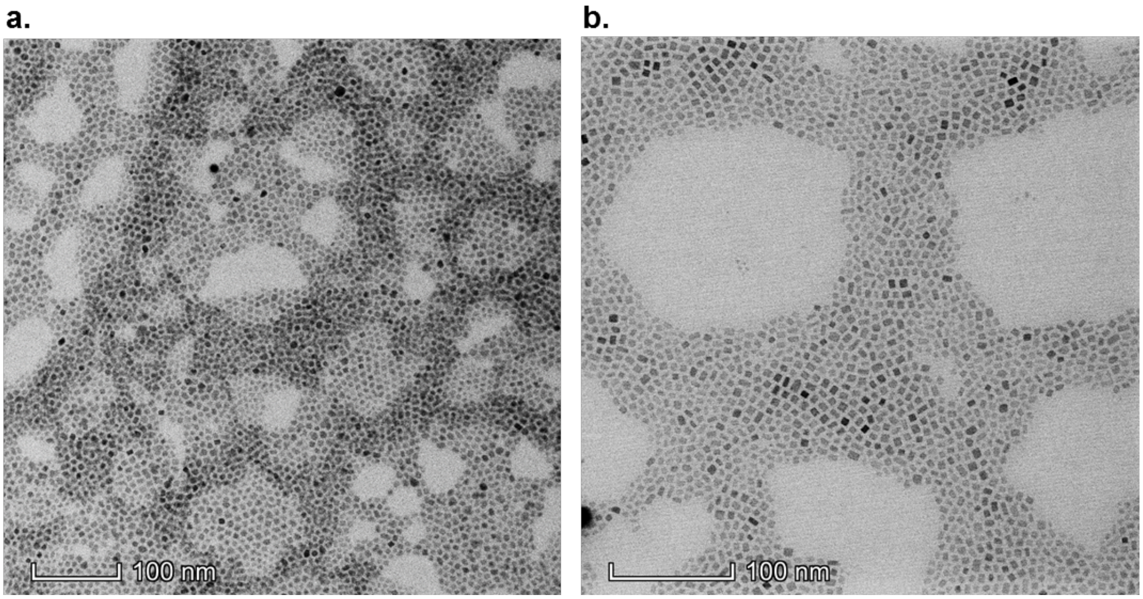

Figure S4. TEM images of OA-Sm:CaF_2 NCs with different amounts of Sm^{3+} dopant. a. 10% Sm:CaF_2 and b. 20%Sm:CaF_2. Contrast variations are related to Bragg diffraction emerging from the crystalline CaF_2 NCs.

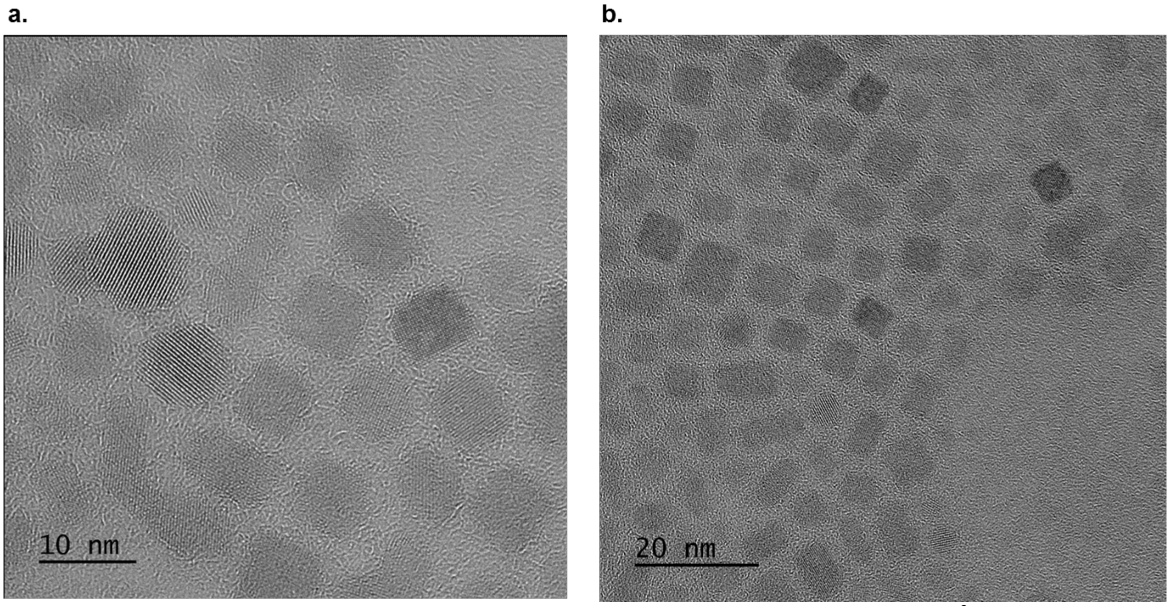

Figure S5. High-resolution TEM images of OA-Sm:CaF_2 NCs with different amounts of Sm^{3+} dopant. a. 10% Sm:CaF_2; b. 20%Sm:CaF_2

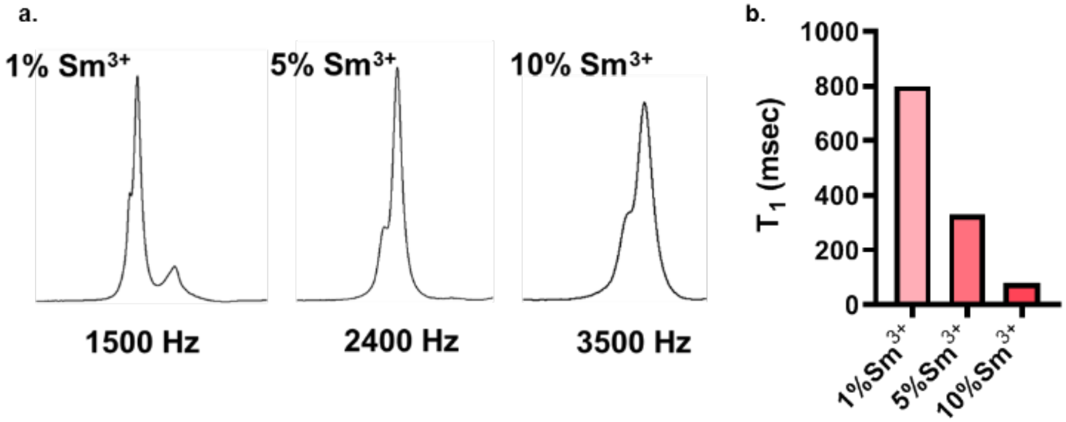

Figure S6. High-resolution ^{19}F-NMR of Sm^{3+}-doped CaF_2 NCs dispersed in cyclohexane. a.^{19}F-NMR spectra of Sm:CaF_2 NCs with different amounts of the dopant (1%, 5% and 10%). b. The calculated T_1 relaxation times of the Sm:CaF_2 NCs shown in (a).

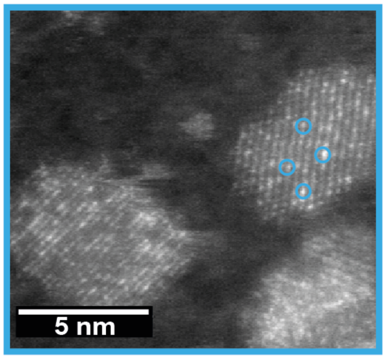

Figure S7. High resolution STEM image of OA-5%Sm:CaF_2. Bright spots are associated with heavy Sm atoms (circled in light-blue in representative locations)

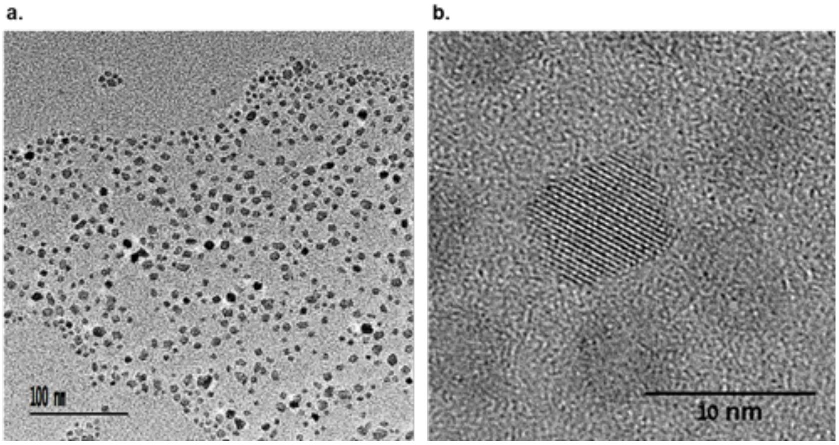

Figure S8. TEM images of water-dispersed PL-Sm:CaF_2. a. TEM and b. HRTEM.

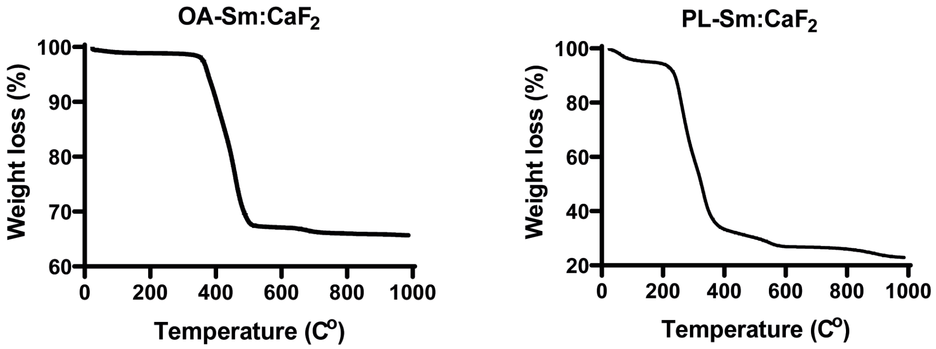

Figure S9. TGA plots for the weight loss of 5%Sm:CaF_2 NCs. a. Coated with OA b. coated with PL.

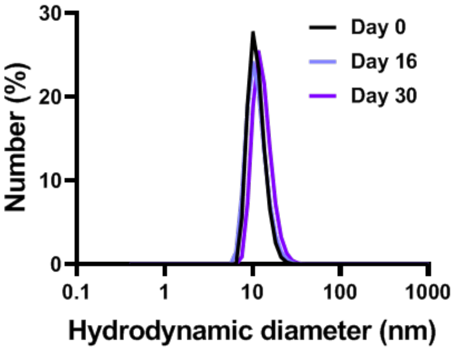

Figure S10. Colloidal stability. DLS measurement for the stability of PL-Sm:CaF_2 NCs, over time in PBS.

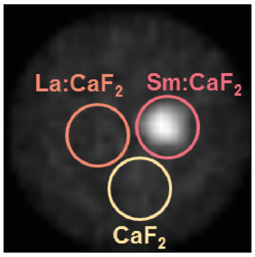

ROI	Sm:CaF_2
Acq. Time	12 sec
SNR	13.95

Figure S11. 3D-UTE- ^{19}F-MRI. Phantom composed of three solutions of PL-CaF_2, PL-La:CaF_2 and PL-Sm:CaF_2 NCs in water. ^{19}F-MR image (left) acquired within 12 sec and the resulted SNR of the PL-Sm:CaF_2 solution.

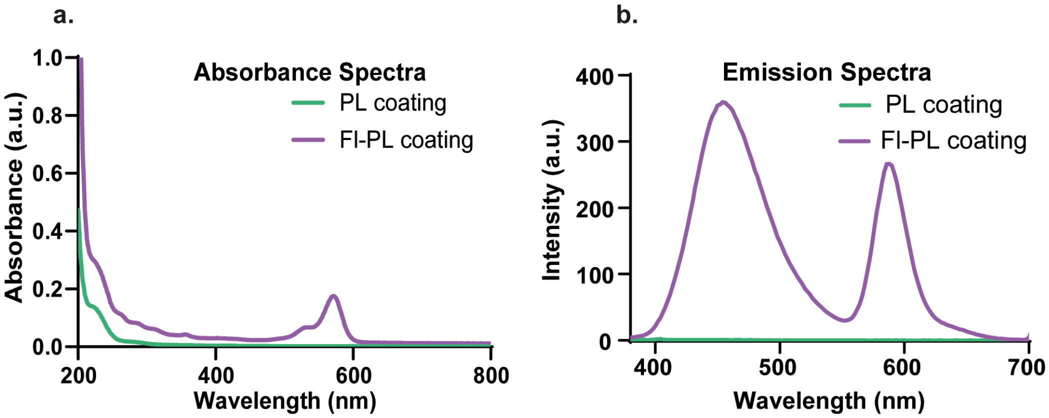

Figure S12. Optical properties of PL-coating NCs. Absorbance (a) and fluorescence (b) spectra of PL-coated (without rhodamine) and FL-PL-coated (with rhodamine) NCs.

Peak name	Ion	MS				
		Molecular formula	m/z<br>MH⁻ ion	Mass, Da		Mass Error, ppm
				Measured	Calculated	
Oleic Acid	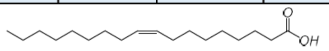<br>Chemical Formula: $C_{18}H_{34}O_2$<br>Exact Mass: 282.2559					
Deprotonated molecular ion	$[M-H]^-$	$C_{18}H_{33}O_2^-$	281	281.2473	281.2481	2.8
PEG1000 PE	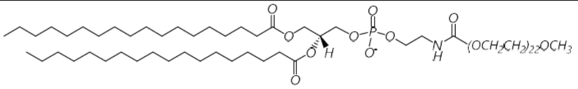<br>Chemical Formula: $C_{87}H_{171}NO_{32}P^-$<br>Exact Mass: 1773.1527					
Deprotonated molecular ion	$[M-H]^-$	$C_{87}H_{171}NO_{32}P^-$	1773	1773.1498	1773.1522	1.4
MHPC	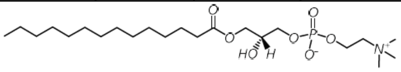<br>Chemical Formula: $C_{22}H_{46}NO_7P$<br>Exact Mass: 467.3012					
Deprotonated molecular ion	$[M-H+Cl]^-$	$C_{22}H_{46}NO_7PCl^-$	502	502.2701	502.2700	0.2

Figure S13. ESI-MS of PL-coated NCs. ESI-MS results from a solution of PL-Sm:CaF_2 NCs verifying the attachment of oleic acid to the CaF_2 NC and the successful incorporation of the phospholipid contents.

a.

Peak name	Ion	MS				
		Molecular formula	m/z<br>MH⁻ ion	Mass, Da		Mass Error, ppm
				Measured	Calculated	
Lactosyl	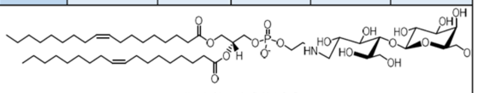<br>Chemical Formula: $C_{53}H_{99}NO_{18}P^-$<br>Exact Mass: 1068.6605					
Protonated molecular ion	$[M-H]^-$	$C_{53}H_{99}NO_{18}P$	1068	1068.6326	1068.6600	>5*

b.

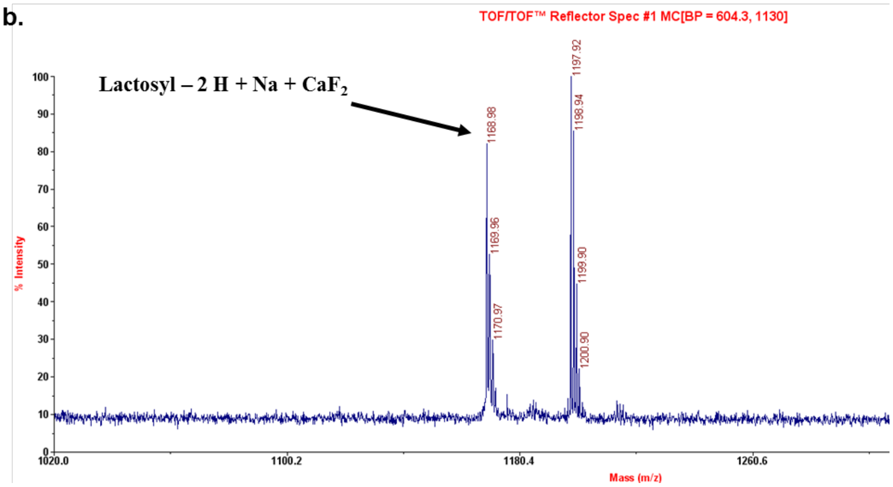

Figure S14. Mass spectrometry analysis of LPL-Sm:CaF_2 NCs. a. ESI and b. MALDI results verifying the lactosyl moiety attached to the NCs.

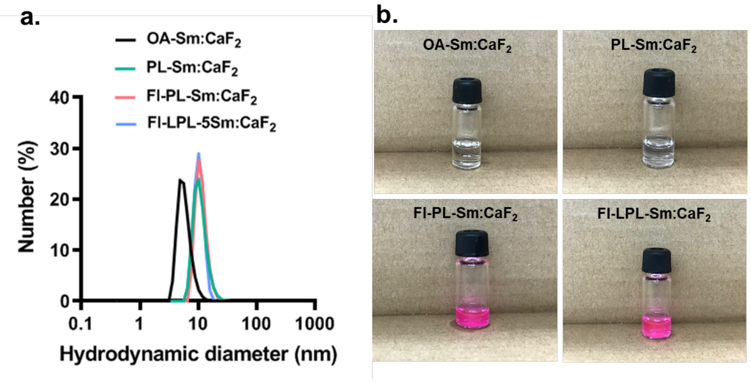

Figure S15. Colloidal size and dispersity of Sm:CaF$_2$ in cyclohexane and water. a. DLS measurement by number of hydrophobic 5%Sm:CaF$_2$ NCs (in cyclohexane) and hydrophilic 5%Sm:CaF$_2$ NCs (in water). b. Pictures of the transparent solutions of hydrophobic (oleate coated, top) and fluorescently labeled-hydrophilic (PL coated, bottom) NCs.

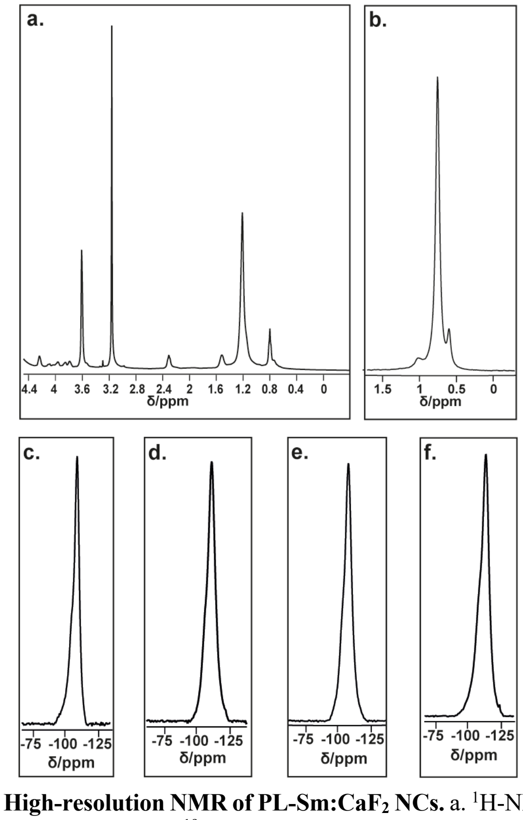

Figure S16: High-resolution NMR of PL-Sm:CaF$_2$ NCs. a. ^{1}H-NMR and b. ^{31}P NMR c. ^{19}F-NMR of hydrophobic OA-Sm:CaF$_2$ NCs in chloroform. d. ^{19}F-NMR of PL-Sm:CaF$_2$ NCs in PBS. e. Fl-PL-Sm:CaF$_2$ NCs in PBS. f. Fl-PL-Lactosyl-Sm:CaF$_2$ NCs. PL: phospholipid; Fl: fluorescently labeled phospholipid.

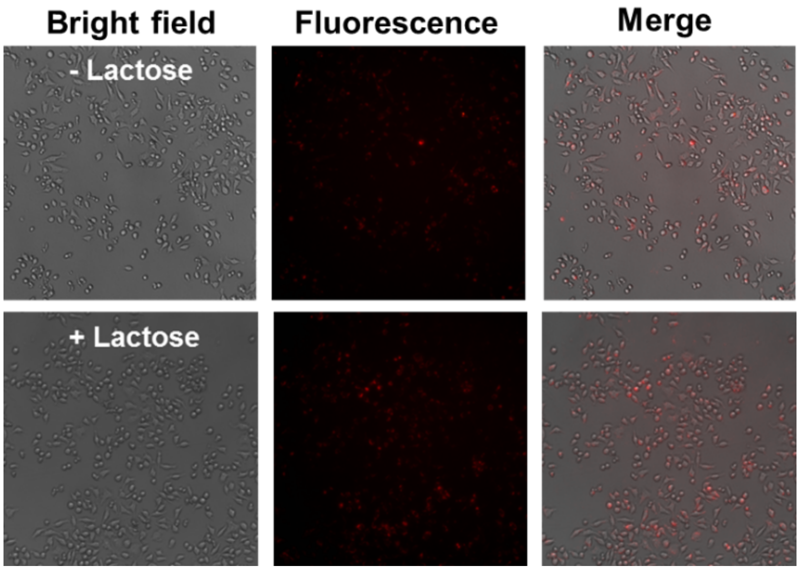

Figure S17. Fluorescent microscopy of RAW264 cells incubated with PL-Sm:CaF_2 and LPL-Sm:CaF_2. Top row: incubation with PL-Sm:CaF_2 NCs. Bottom row, incubation with LPL-Sm:CaF_2 NCs.

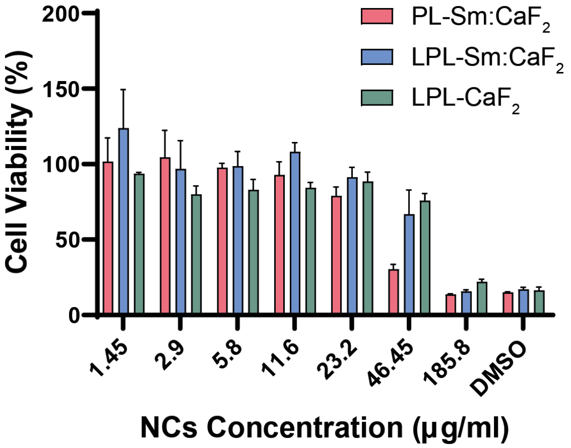

Figure S18. Cell viability assay of RAW264 cells. Bar graph comparing three different compositions of phospholipid-coated NCs, PL-Sm:CaF_2 (no lactose with Sm^{3+} dopant), LPL-Sm:CaF_2 (with lactose and with Sm^{3+} dopant) and LPL-CaF_2 (with lactose and without Sm^{3+}), which were incubated with RAW264 cells with increasing concentration of NCs (from left to right). The cells' viability was calculated relative to 100% viability (i.e., cells not treated with NCs). Cells treated with 50% DMSO were used as positive control. Cell viability was evaluated using the CCK-8 assay.

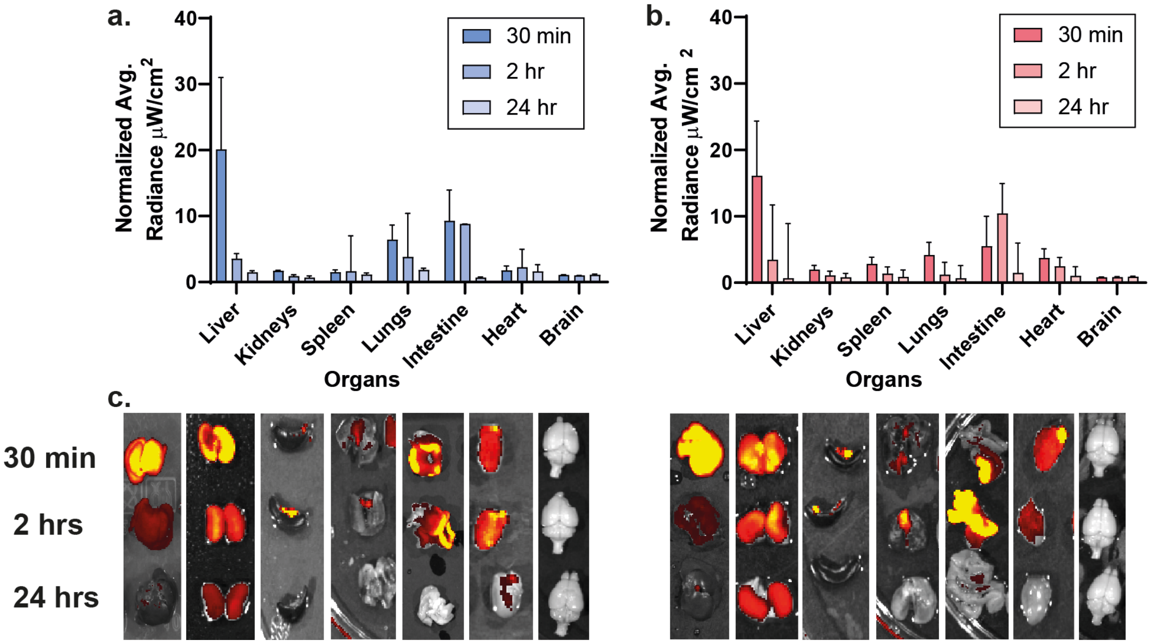

Figure S19. Time-dependent biodistribution profiles of LPL-Sm:CaF_2 vs. PL-Sm:CaF_2 NCs. Bar graph showing the normalized average radiance (μW/cm^2) of the fluorescence signal (rhodamine) of different organs that were harvested from two groups of mice that were intravenously injected with either LPL-Sm:CaF_2 NCs (a) or PL-Sm:CaF_2 NCs (b) 30 min (N=3 for each group), 2 hr (N=3 for each group) and 24 hr (N=3 for each group) following their administration. c. Representative fluorescence images of excised organs at different time points following the NCs' administration (left, LPL-Sm:CaF_2; right, PL-Sm:CaF_2). NCs were injected intravenously as 50μL solution of 25 mg/mL NCs in PBS.

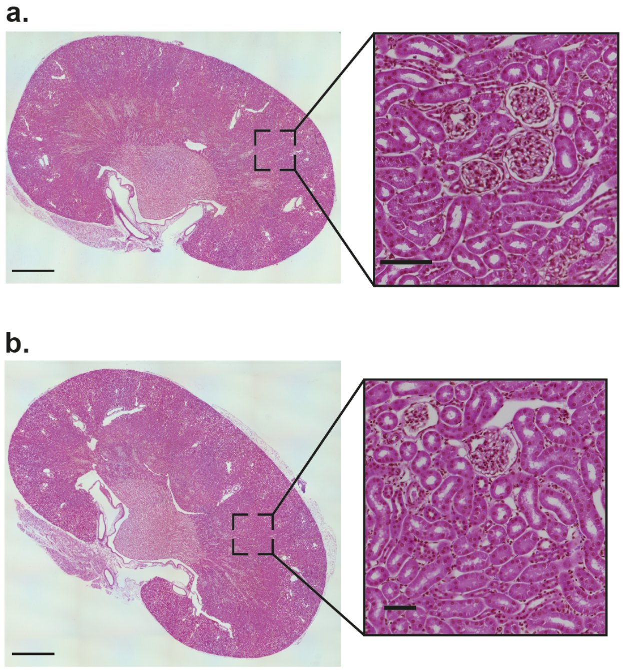

Figure S20. H&E staining of mice kidneys. Shown are micrograph images (left) of kidneys excised 24 hr after LPL-Sm:CaF_2 NC (a) or PBS (control; b) injection; scale bar: 1000 μm. Enlarged areas (right) demonstrate the absence of kidney damage; scale bar: 100 μm.

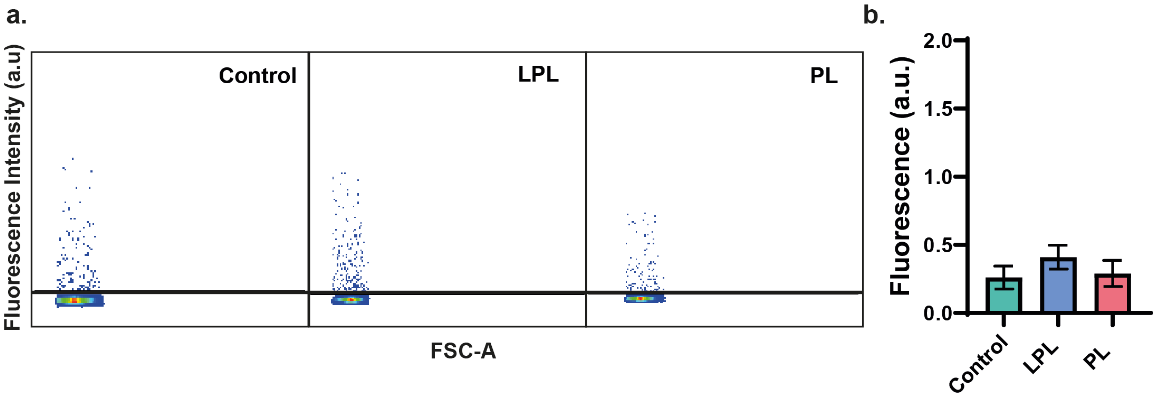

Figure S21. ***In-vivo*** **toxicity evaluation of LPL-Sm:CaF_2 NCs vs. PL-Sm:CaF_2 NCs and control (in PBS).** a. Representative FACS dot plots of cells (stained with DAPI for dead cell representation) excised from lymph nodes of inflamed mice 2 hr post injection of PBS (control, left), LPL-Sm:CaF_2 (middle, labeled LPL) or PL-Sm:CaF_2 (right, labeled PL). NCs were injected as 20μL solution of 75 mg/mL NCs in PBS. b. Quantitative analysis of the FACS data (DAPI) obtained from three different mice (N=3). Cells were stained with DAPI to determine the toxicity of the injected NCs.

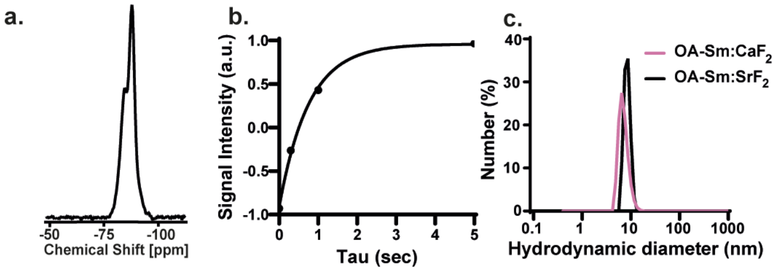

Figure S22. OA-Sm:SrF_2. a. high resolution ^{19}F-NMR of OA-Sm:SrF_2 suspended in cyclohexane , δ = -88 ppm. b. Inversion recovery plot used to determine the T_1 relaxation times (770 msec) of Sm:SrF_2. c. DLS histograms of dispersed OA-Sm:SrF_2 and OA-Sm:CaF_2 .

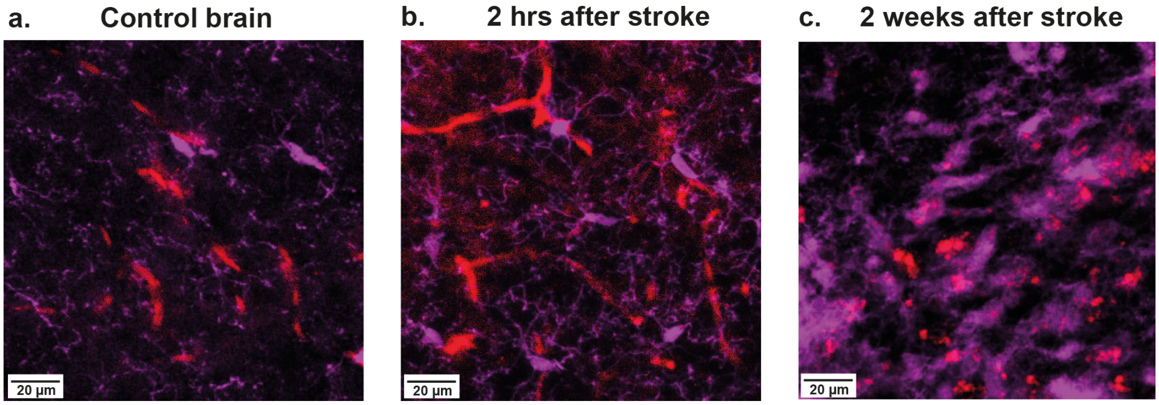

Figure S23. Confocal fluorescent microscopy brains' sections of mice was injected with 70 kDa dextran-red. Single slice of confocal sections, at 20x magnification of a. Control brain (no stroke). b. Two hours after stroke induction. c. Two weeks after stroke induction. Iba-1 expressing cells shown in magenta and dextran in red.

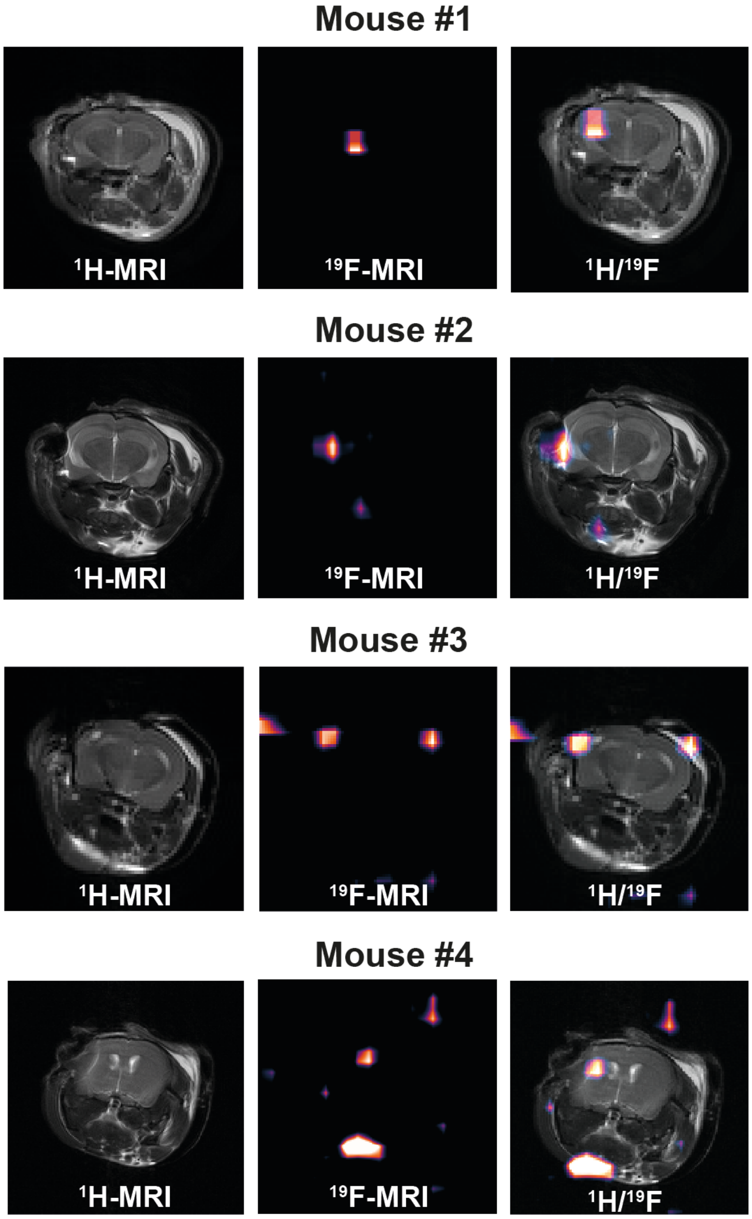

Figure S24. *In-vivo* MRI of neuroinflammation of four different mice. Left- *in-vivo* ^{1}H-MRI of a mouse brain two weeks after stroke induction (left hemisphere), middle – ^{19}F signal of UTE-3D images acquired on the working frequency of LPL-Sm:CaF_2 (δ = -109 ppm), right - overlay presentation of the ^{1}H/^{19}F images. MRI data was acquired 2 hr after LPL-Sm:CaF_2 injection. Note here that the additional ^{19}F-MRI signals observed in mice #2 and #4 are from the lymph nodes in the head of the mice. For mouse #3, the additional ^{19}F-MRI signal (out of the infarct region) might have arisen from residual LPL-Sm:CaF_2 in the blood. The LPL-Sm:CaF_2 were injected retro-orbitally contralaterally to the stroke region.

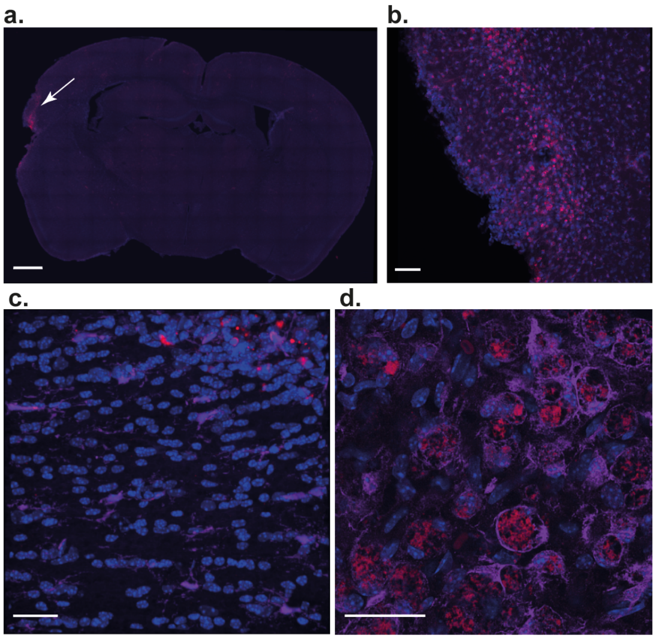

Figure S25. Confocal fluorescent microscopy of mouse brain. Fluorescent images of cryo-sectioned brain slices obtained two weeks after stroke induction and 2 hrs following retro-orbitally injection of LPL-Sm:CaF_2. a. single slice of confocal sections, at 20x magnification, of the whole brain with white arrow pointing the stroke region. b. Stack of confocal sections, at 20x magnification, of the region of the stroke showing massive accumulation of the rhodamine-labeled LPL-Sm:CaF_2 in red. Single confocal virtual plane at 63x magnification of contralateral (c) and ipsilateral (d) regions. Cell nuclei are stained with DAPI (blue), LPL-Sm:CaF_2 are stained with rhodamine (red) and Iba-1 expressing cells shown in magenta. Scale bar : 30 µm.

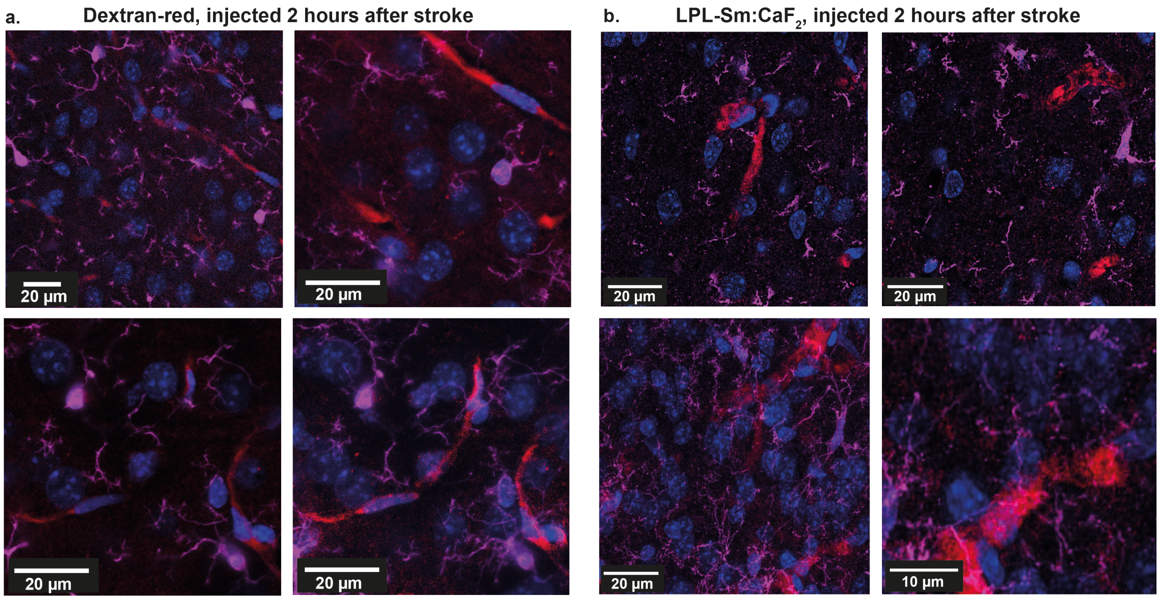

Figure S26. Confocal fluorescent microscopy of a mouse brain 2 hours after stroke induction. Fluorescent images of cryo-sectioned brain slices obtained two hours after stroke induction, following retro-orbitally injection of (a) dextran-red (70 kDa) or (b) LPL-Sm:CaF_2. Cell nuclei are stained with DAPI (blue), LPL-Sm:CaF_2 are labeled with rhodamine, dextran with red and Iba-1-expressing cells are shown in magenta

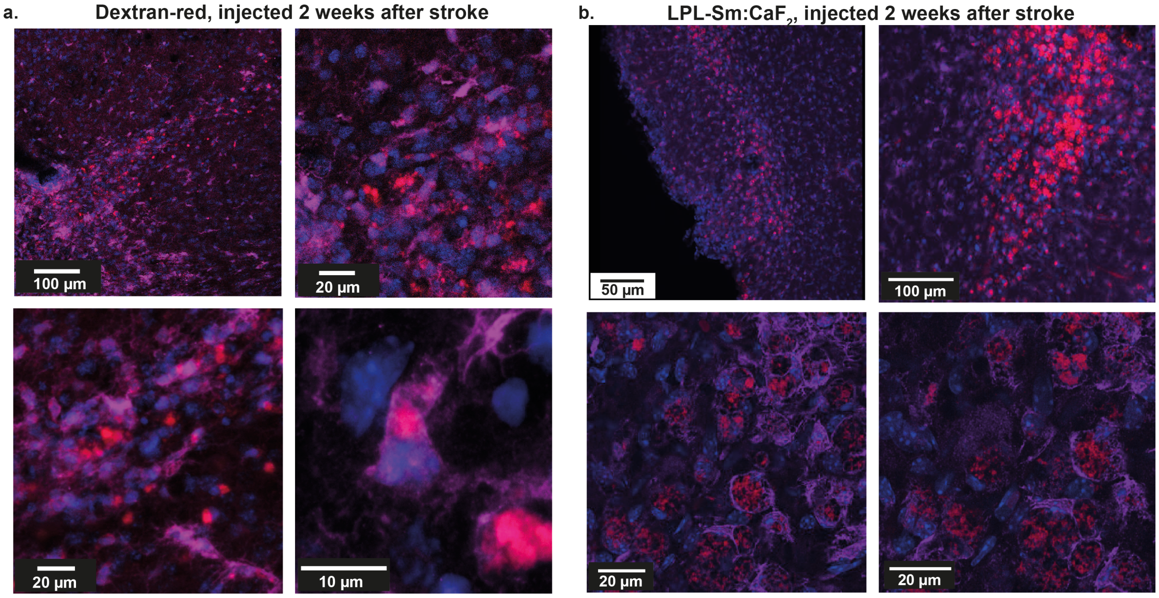

Figure S27. Confocal fluorescent microscopy of a mouse brain two weeks after a stroke. Fluorescent images of cryo-sectioned brain slices obtained two weeks after stroke induction, following retro-orbitally injection of (a) dextran-red (70 kDa) or (b) LPL-Sm:CaF_2. Cell nuclei are stained with DAPI (blue), LPL-Sm:CaF_2 are labeled with rhodamine, dextran with red and Iba-1-expressing cells are shown in magenta.

Supporting Tables

Table S1. List of the different phospholipids (PLs) that were used for the synthesis of either PL or LPL – Sm:CaF_2 NCs.

Compound	Mw (g/mol)	Weight (mg)	n (mmol)
Lyso-PC	467.57	30	0.064
PEG-PE	1792.28	5	$2.78*10^{-3}$
Cholesterol	386.65	2	$5.17*10^{-3}$
Rhod-PE	1192.61	0.195	$1.63*10^{-4}$
**Lactosyl-PE	1087.36	0.302	$2.78*10^{-4}$

**10% mol of PEG-PE were subtituted by Lactosyl-PE

Table S2. T_1 relaxation times that were measured at different magnetic fields (B_0) a. PL-Sm:CaF_2 and b. LPL-Sm:CaF_2

a.

Magnetic field (T)	T_1 (ms)
**7.0**	65
**9.4**	70
**11.7**	72

b.

Magnetic field (T)	T_1 (ms)
**7.0**	65
**9.4**	72
**11.7**	75

Table S3. Contrast-to-noise ratio (CNR) calculation from a "multicolor" phantom (Figure 6c). CNR was evaluated by dividing the SNR obtained from each tube (containing either CaF_2 or SrF_2) at each one of the studied frequencies (-109 or -88 ppm).

NCs' type / Chemical shift (ppm)	CNR (a.u)
SrF_2 / - 88	9.133
CaF_2 / -88	0.109
SrF_2 / -109	0.107
CaF_2 / -109	9.302

Table S4. Dehydration protocol of kidney immunostaining

Step No.	Reagent	Step Duration (min)
1	Ethanol 70%	45
2	Ethanol 95%	45
3	Ethanol 95%	45
4	Ethanol 95%	45
5	Ethanol 100% (Abs.)	30
6	Ethanol 100% (Abs.)	30
7	1:1 (Abs./Solvent)	45
8	Xylene	60
9	Xylene	60
10	Wax (57◦C)	60
11	Wax (57◦C)	60
12	Wax (57◦C)	60

Table S5. Protocol timings used for kidney H&E staining

Step No.	Reagent	Step Duration (min)
1	Xylene	5
2	Xylene	5
3	Xylene	5
4	Ethanol 100% (Abs.)	5
5	Ethanol 95%	5
6	Ethanol 70%	5
7	TP (Tap Water)	1
8	DW (Distilled Water)	1
9	Hematoxylin	5
10	TW	2
11	Acid Alcohol**	*3 sec
12	TW	3
13	TW	3
14	DW	1
15	Ethanol 70%	3
16	eosin	1
17	Ethanol 95%	2
18	Ethanol 95%	2
19	Ethanol 100% (Abs.)	3
20	Ethanol 100% (Abs.)	3
21	Xylene	3
22	Xylene	3

** 500 ml Ethanol 70% + 3ml HCl
